# Effects of inspiratory muscle training on inspiratory muscle strength and exercise tolerance in patients with COPD: a meta-analysis and systematic review

**DOI:** 10.3389/fmed.2026.1855676

**Published:** 2026-06-29

**Authors:** Lei Xu, Qi Liao

**Affiliations:** Department of General Medicine, Panzhihua Central Hospital, Panzhihua, China

**Keywords:** 6-min walk distance, chronic obstructive pulmonary disease, inspiratory muscle training, respiratory muscle training, systematic review

## Abstract

**Objective:**

To systematically evaluate the effects of inspiratory muscle training (IMT) on inspiratory muscle strength, exercise tolerance, and dyspnea in patients with COPD, including both stable and post-AECOPD rehabilitation-phase participants, and to explore the moderating role of comparator type on treatment efficacy.

**Methods:**

Following PRISMA 2020 guidelines, we systematically searched PubMed, Embase, Cochrane Library, Web of Science, and Scopus from inception to March 1, 2026. Randomized controlled trials (RCTs) involving adults with stable COPD were included. The primary outcomes were PImax, 6MWD, and dyspnea (SMD). A random-effects model was used to pool effect sizes, and subgroup analyses, sensitivity analyses, and meta-regression were performed.

**Results:**

Thirteen RCTs (*n* = 1,170) were included. IMT significantly improved PImax (SMD = 1.23, 95%CI 0.12–2.34, *p* = 0.034, *I*^2^ = 90.9%) and 6MWD (SMD = 0.43, 95%CI 0.09–0.77, *p* = 0.018, *I*^2^ = 70.6%). The improvement in 6MWD reached statistical significance only in the sham-controlled subgroup (SMD = 0.51, *p* = 0.046), whereas no additional benefit was observed in the “IMT added to pulmonary rehabilitation (PR)” subgroup (SMD = −0.05, *p* = 0.875). Dyspnea showed a favorable trend that did not reach statistical significance (SMD = 0.33, 95%CI −0.11–0.77, *p* = 0.129), and no significant improvement was found in FEV₁ (MD = 0.14 L, 95%CI −0.17–0.44, *p* = 0.190). Meta-regression suggested that comparator type explained approximately 37.8% of the heterogeneity in 6MWD (*p* = 0.062).

**Conclusion:**

IMT can significantly enhance inspiratory muscle strength and improve exercise tolerance, but its incremental benefit is highly dependent on the comparator type. The effect is more evident in non-pulmonary rehabilitation control settings, whereas additional benefit is limited in patients who have already undergone comprehensive pulmonary rehabilitation. IMT is more suitable for stable COPD patients who cannot tolerate standard pulmonary rehabilitation. Future high-quality, longer-term head-to-head studies are needed to determine the optimal strategies for different training modalities.

**Systematic review registration:**

https://doi.org/10.37766/inplasy2026.5.0046, identifier (INPLASY202650046).

## 1 Introduction

A common yet preventable and treatable chronic respiratory disease, COPD is characterized by persistent respiratory symptoms and airflow restriction. These features severely impair the health status and quality of life of affected individuals (1). Even during the stable phase, many COPD patients continue to experience dyspnea, activity limitation, and reduced health-related quality of life, which not only restrict daily function but also increase the complexity of disease management and healthcare burden (2, 3). Therefore, exploring effective and feasible non-pharmacological interventions for stable COPD patients has important clinical implications for symptom control and rehabilitation outcomes.

Due to airflow limitation, dynamic lung hyperinflation, and increased work of breathing, COPD patients often have increased respiratory muscle (especially inspiratory muscle) load and impaired function. This is considered one of the important pathophysiological bases for dyspnea and reduced exercise tolerance (4, 5). Inspiratory muscle training (IMT), as an important component of pulmonary rehabilitation, aims to enhance inspiratory muscle strength and endurance, improve respiratory mechanics efficiency, and reduce relative ventilatory load, thereby alleviating dyspnea and improving functional status (6, 7). In recent years, respiratory muscle training has been increasingly applied in COPD rehabilitation practice, and its potential benefits have gained increasing attention.

Several randomized controlled trials and systematic reviews have evaluated the effects of respiratory muscle training in COPD patients. Some studies have shown that respiratory muscle training, especially IMT, may improve dyspnea, maximal inspiratory pressure, and some quality-of-life indicators (8). However, the existing evidence is not entirely consistent, and there is considerable heterogeneity across studies in terms of population, disease stage, training type, load intensity, frequency, duration, and whether combined with pulmonary rehabilitation (9–11). Moreover, previous systematic reviews have different inclusion times and analytical focuses; some have mixed COPD patients at different clinical stages, which may increase clinical heterogeneity and affect the interpretation and generalizability of results. Notably, most existing systematic reviews have analyzed respiratory muscle training as a homogeneous intervention and often mixed patients at different COPD stages with diverse control conditions, potentially masking the true efficacy differences of IMT under different control strategies, thus limiting the clinical interpretability and practical guidance value of the results. A recent systematic review suggested that RMT may improve dyspnea and quality of life in COPD patients, but the existing evidence often pools various forms of breathing exercises and respiratory muscle training, and the heterogeneity in populations and intervention protocols limits the assessment of the clinical value of different training modalities (12).

Therefore, this study aims to systematically evaluate the effects of IMT exclusively on respiratory muscle strength, exercise tolerance, and dyspnea in stable COPD patients. While previous reviews often pooled multiple respiratory muscle training (RMT) modalities including EMT or combined interventions, we explicitly restricted the intervention scope to IMT to minimize clinical heterogeneity and focus on the targeted effects of inspiratory resistance training. Studies employing EMT alone or complex combined RMT were excluded unless they clearly reported an IMT-specific arm. Subgroup analyses based on comparator type (e.g., sham, usual care/no intervention, background-matched add-on) were implemented to isolate the true benefits of IMT in different clinical contexts, providing precise evidence-based guidance for individualized rehabilitation of stable COPD patients.

## 2 Methods

### 2.1 Study design

The present investigation takes the form of a systematic review and meta-analysis, with the objective of assessing how IMT influences inspiratory muscle strength, exercise tolerance, dyspnea, and lung function in patients with COPD, including both stable-phase and post-AECOPD rehabilitation-phase participants. Its design, execution, and reporting are aligned with the PRISMA 2020 statement (Supplementary Table S1). The study protocol was retrospectively registered in INPLASY on May 6, 2026, after data extraction had already begun (registration no. INPLASY202650046, DOI: 10.37766/inplasy2026.5.0046). This retrospective registration was intended to enhance transparency by documenting the eligibility criteria, outcomes, and analysis plan used in the review; however, it should not be interpreted as prospective pre-specification. Therefore, this review cannot be considered prospectively registered. The comparator-based subgroup analyses and meta-regression should be interpreted as exploratory, and the retrospective registration may introduce potential risk of selective reporting (Table 1).

**Table 1:** Eligibility criteria.

Element	Definition
P	Adults aged ≥18 years diagnosed with COPD (GOLD I–IV); stable phase (≥4 weeks after AECOPD resolution) or post-AECOPD rehabilitation phase (intervention initiated ≤2 weeks after hospital discharge)
I	Defined inspiratory muscle training (IMT) only: using threshold/resistive devices (e.g., POWERbreathe, eBreather); intensity ≥30% PImax (or individually adjusted); frequency ≥3 times/week, total duration ≥3 weeks. Studies employing EMT alone or combined RMT interventions were excluded unless an IMT-specific arm could be clearly identified. IMT was considered the sole core intervention for eligibility
C	Comparator must be: sham IMT; PR alone; usual care/health education; other non-IMT training (e.g., ET alone). Excluded: no control, cross-sectional only, mechanistic observational studies
O	1. Maximal inspiratory pressure (PImax/MIP, cmH₂O); 2. 6-min walk distance (6MWD, m); 3. Dyspnea measured using Borg, mMRC, TDI, or comparable scales and pooled as standardized mean difference (SMD); 4. FEV₁ reported in liters (L) for quantitative synthesis, with % predicted values used for eligibility verification and descriptive assessment of disease severity when available.
S	Randomized controlled trials (RCTs), including parallel, cross-over, multi-center designs; non-RCTs used only for narrative analysis

### 2.2 Eligibility criteria

Exclusion criteria: acute exacerbation or hospitalized/ICU/mechanically ventilated patients; non-COPD or mixed disease populations where COPD data cannot be extracted; intervention not structured respiratory muscle training; non-randomized studies, reviews, case reports, animal studies; severely missing data that cannot be obtained.

### 2.3 Search strategy

A literature search covering the period from database inception to March 1, 2026 was carried out across PubMed, Embase, Cochrane Library, Web of Science, and Scopus. Additional records were sought from ClinicalTrials.gov and the WHO ICTRP. As a supplementary strategy, we manually scanned the reference lists of the included studies and of relevant systematic reviews to capture any potentially eligible articles not identified through the primary search.

The search strategy was built around three core concepts: COPD, respiratory muscle training (including inspiratory and expiratory muscle training), and randomized controlled trials. Both controlled vocabulary (MeSH) and free-text terms were used accordingly. Below is an illustrative search string applied in PubMed: (“Pulmonary Disease, Chronic Obstructive” [Mesh] OR COPD OR chronic obstructive pulmonary disease) AND (“Respiratory Muscles” [Mesh] OR “respiratory muscle training” OR “inspiratory muscle training” OR IMT OR “expiratory muscle training” OR EMT OR “ventilatory muscle training”) AND (randomized controlled trial[Publication Type] OR randomized OR randomised OR randomly OR trial).

The same search principles were adapted for each of the other databases according to their specific indexing rules and syntax.

### 2.4 Study selection

Imported records were deduplicated in EndNote. Based on the predetermined eligibility criteria, two reviewers conducted a two-phase selection independently. Phase one: screening titles/abstracts to eliminate obviously unrelated studies. Phase two: assessing full texts of the remaining articles for final inclusion. Disagreements between reviewers were resolved via discussion, or by a third reviewer if necessary. A PRISMA flow diagram outlines the whole selection process, and documented reasons are provided for each full-text exclusion.

### 2.5 Data extraction

Two reviewers independently extracted information using a pre-designed data extraction form, including: (1) Basic information: first author, year of publication, country/region; (2) Study characteristics: study design, randomization method, sample size, follow-up duration; (3) Participant characteristics: mean age, sex distribution, COPD severity; (4) Intervention characteristics: intervention type (IMT), whether combined with pulmonary rehabilitation, type of training device, training intensity, frequency, session duration, total training period, supervision method (supervised/home-based); (5) Comparator characteristics: usual care, sham training, PR alone, etc.; (6) Outcome data: mean, standard deviation, sample size at each time point, or data that could be converted to effect sizes; (7) Risk of bias-related information: allocation concealment, blinding, missing data, completeness of outcome reporting, etc.; (8) Other information: funding source, conflicts of interest.

If multiple follow-up time points were reported, we preferentially extracted data assessed immediately after the intervention for the main analysis; long-term follow-up data were used for supplementary analyses. If data were missing, we attempted to contact the corresponding author to obtain them.

### 2.6 Outcome definitions

If a study reported multiple time points, we preferentially used the following order: (1) at intervention end; (2) the time point closest to intervention end; for long-term follow-up analyses, we extracted follow-up data separately and did not mix them with post-intervention data.

If a study used multiple scales for the same outcome, we extracted data according to the following hierarchy. For dyspnea, we first distinguished dyspnea during daily living from exercise-induced dyspnea. When multiple dyspnea outcomes were reported in the same study, we selected one outcome according to clinical relevance and comparability across studies, prioritizing daily-life dyspnea over exercise-induced dyspnea and TDI when they reflected the same assessment context. If multiple dyspnea scales measured the same construct at the same time point and could not be clearly prioritized, they were combined into a single study-level effect size to avoid double counting. All dyspnea scales were oriented so that a positive SMD indicated improvement. For scales in which lower scores indicated less dyspnea, such as Borg and mMRC, the sign of the effect estimate was reversed when necessary; for scales in which higher scores indicated improvement, such as TDI, the original direction was retained. For inspiratory muscle strength, we preferentially used absolute values in cmH₂O for PImax/MIP. For FEV_1_, values reported in liters were extracted for quantitative pooling to ensure consistency across studies, whereas % predicted values were used only for eligibility verification or descriptive characterization of baseline lung-function impairment. If studies reported standard errors, 95% confidence intervals, medians with interquartile ranges, etc., we converted them following Cochrane Handbook recommendations. When necessary, data were extracted from graphs and verified by two reviewers.

### 2.7 Risk of bias assessment

Two reviewers independently used the Cochrane RoB 2.0 tool to appraise the methodological quality of the included RCTs. The assessment covered five domains: bias arising from the randomization process; bias due to deviations from intended interventions; bias due to missing outcome data; bias in measurement of the outcome; and bias in selection of the reported result. Each domain was judged as “low risk,” “some concerns,” or “high risk,” and an overall risk-of-bias judgment was generated for each study. Disagreements were resolved through discussion or, if necessary, by a third reviewer. The use of RoB 2.0 reflects current best practice since 2019, providing a more structured and outcome-specific assessment compared with RoB 1.0.

### 2.8 Statistical analysis

For continuous variables, if studies used the same unit and scale, we used the mean difference (MD) with 95% confidence interval (CI); if different studies used different scales to assess the same conceptual outcome, we used the standardized mean difference (SMD) with 95% CI. For dyspnea outcomes, SMDs were harmonized so that positive values consistently represented improvement after IMT. For FEV_1_, MDs were calculated in liters because this was the most consistently extractable unit across the included trials. We preferentially extracted change scores from baseline to intervention end; if change scores were not available, we used post-intervention endpoint values for pooling. Continuous variables were extracted as mean ± standard deviation whenever possible; categorical variables were extracted as counts or proportions. For studies that reported only medians with interquartile ranges, ranges, or confidence intervals without means and standard deviations, we converted them to approximate means and standard deviations using accepted statistical methods. Change scores from baseline to the end of intervention were preferentially used because they account for baseline imbalance. When change scores were unavailable, post-intervention endpoint values were extracted. Because several outcomes included both change-score and endpoint-value data, the primary analyses used SMDs where appropriate, and separate supplementary MD analyses were conducted for change-score studies and endpoint-value studies to examine the robustness of pooling decisions.

Heterogeneity across studies was assessed using the Cochran Q test and *I*^2^ statistic: *I*^2^ < 25%: low heterogeneity; 25% ≤ *I*^2^ < 50%: moderate heterogeneity; *I*^2^ ≥ 50%: substantial heterogeneity. Considering potential clinical heterogeneity in population characteristics, training modalities, intervention intensity, and control conditions across studies, we preferentially used a random-effects model for meta-analysis; when heterogeneity was very low, we also reported fixed-effect model results as a supplement.

Exploratory subgroup analyses were conducted according to intervention strategy, training duration, supervision method, and comparator type: (1) intervention strategy: IMT alone versus IMT combined with PR/exercise; (2) training duration: <8 weeks, 8–12 weeks, and >12 weeks; (3) supervision method: supervised versus unsupervised/home-based; and (4) comparator type: inactive control, including no training, usual care, or sham training, versus active control, such as PR alone. Studies with EMT alone or combined RMT were not pooled unless an IMT-specific arm was clearly reported, ensuring that all pooled analyses reflected the effects of inspiratory muscle training only. For multi-arm trials, only eligible IMT and relevant comparator arms were included. When more than one eligible IMT arm shared the same comparator group, the comparator sample size was divided according to Cochrane Handbook recommendations to avoid double counting. When different IMT modalities within the same trial were conceptually similar and clinically appropriate to combine, their means, standard deviations, and sample sizes were pooled into a single IMT group before meta-analysis.

## 3 Results

### 3.1 Study selection process

**Figure 1:**
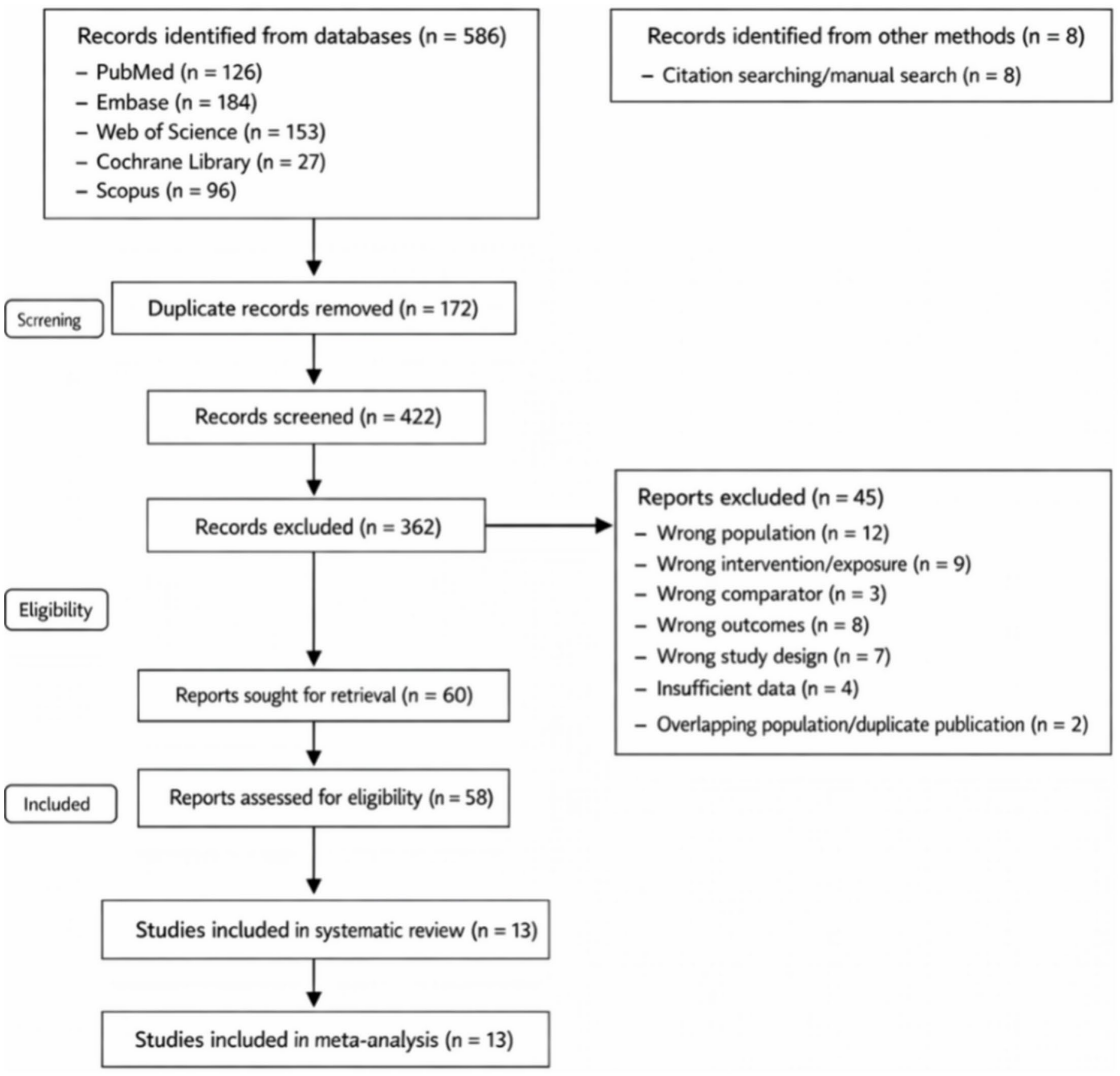
PRISMA flow diagram.

The database search yielded 586 records: PubMed 126, Embase 184, Web of Science 153, Cochrane Library 27, Scopus 96; an additional 8 records were obtained through citation tracking/manual searching. After removing 172 duplicates, 422 records entered title and abstract screening, of which 362 were excluded. Full texts were sought for 60 records, and 2 could not be obtained. Finally, 58 full-text articles were assessed for eligibility, of which 45 were excluded for reasons including: ineligible study population (12), ineligible intervention/exposure (9), ineligible comparator (3), ineligible outcome (8), ineligible study design (7), insufficient data (4), and overlapping populations/duplicate publications (2). Thirteen studies were finally included in the systematic review, and all 13 were included in the meta-analysis (Figure 1).

### 3.2 Basic characteristics of included studies

A total of 13 randomized controlled trials, published between 2015 and 2026, were included, covering Europe, Asia, and South America. Most studies used a parallel-group RCT design, some with double-blind, single-blind, or sham-controlled designs, and some were multi-arm trials. Sample sizes ranged from 16 to 602, intervention durations ranged from 3 to 24 weeks, with most studies having a training period of 8 weeks. Participants were predominantly middle-aged and elderly COPD patients, with disease severity mainly GOLD II–IV. Most studies enrolled clinically stable COPD patients, whereas one study enrolled participants in the post-AECOPD rehabilitation phase. This mixed population was retained according to the eligibility criteria, but its potential contribution to clinical heterogeneity was considered in interpretation and sensitivity analyses. Interventions included IMT alone as well as IMT combined with pulmonary rehabilitation or exercise training. Training devices were mostly threshold-loaded devices such as Threshold IMT or POWERbreathe, with training intensity typically set at 30–60% PImax/MIP and progressively increased. Overall, the included studies showed some clinical heterogeneity in population characteristics, intervention protocols, and supervision methods (Table 2).

**Table 2:** Population characteristics and intervention protocols of included studies.

Study	Country/Region	Design	Sample size	COPD severity	Intervention	Combined with PR/ET	Training protocol	Duration
Beaumont 2015 (13)	France	Parallel RCT	34	GOLD 1–4	IMT vs. PR	Yes	40% PImax, 2/day	3 weeks
Chuang 2017 (14)	Taiwan, China	RCT	55	GOLD II–IV	Threshold IMT vs. control	No	Progressive load, 5 days/week	8 weeks
Langer 2018 (15)	Canada/Belgium	Double-blind RCT	20	Predominantly GOLD 3–4	IMT vs. sham	No	40–50% PImax	8 weeks
Xu 2018 (16)	Guangzhou, China	Four-arm RCT	87	GOLD I–IV	Sham/IMT/combined training	No	30% → 45% PImax	8 weeks
Schultz 2018 (17)	Germany	Parallel RCT	602	GOLD II–IV	IMT vs. sham	Yes	30% → 68.5% PImax	3 weeks
Beaumont 2018 (18)	France	Single-blind RCT	149	Severe/very severe	PR + IMT vs. PR	Yes	50% → 60% PImax	4 weeks
Cutrim 2019 (19)	Brazil	Single-center RCT	22	GOLD A, I–II	IMT vs. control	No	30% PImax, 3/week	12 weeks
Saka 2021 (20)	Turkey	Sham-controlled RCT	40	Predominantly GOLD III–IV	IMT vs. sham	No	30% MIP, 2/day	8 weeks
Tounsi 2021 (21)	Tunisia/France	Parallel RCT	32	GOLD II–IV	IMT + ET vs. ET	Yes	50% → 80% PImax	8 weeks
Wu 2024 (22)	China	Open-label RCT	75	Moderate to very severe	R-IMT/T-IMT/control	No	60% PTPMIP, 2/day	8 weeks
Dosbaba 2025 (23)	Czech Republic	Three-arm RCT	36	GOLD II–III	TIRE/Threshold/sham	No	1/day	24 weeks
Elsayed 2026 (24)	Egypt	Double-blind RCT	60	FEV₁/FVC ≤ 70%	IMT + aerobic exercise	Yes	2/day	4 weeks
Huang 2025 (25)	Taiwan, China	Double-blind RCT	16	Post-AECOPD recovery	IMT + PR vs. PR	Yes	30% PImax, 7 days/week	8 weeks

Sample sizes reported refer to the number of participants included in the meta-analysis from each study. For multi-arm trials, only eligible IMT and relevant comparator arms were included, and shared comparator groups were split or combined according to Cochrane recommendations.

**Figure 2:**
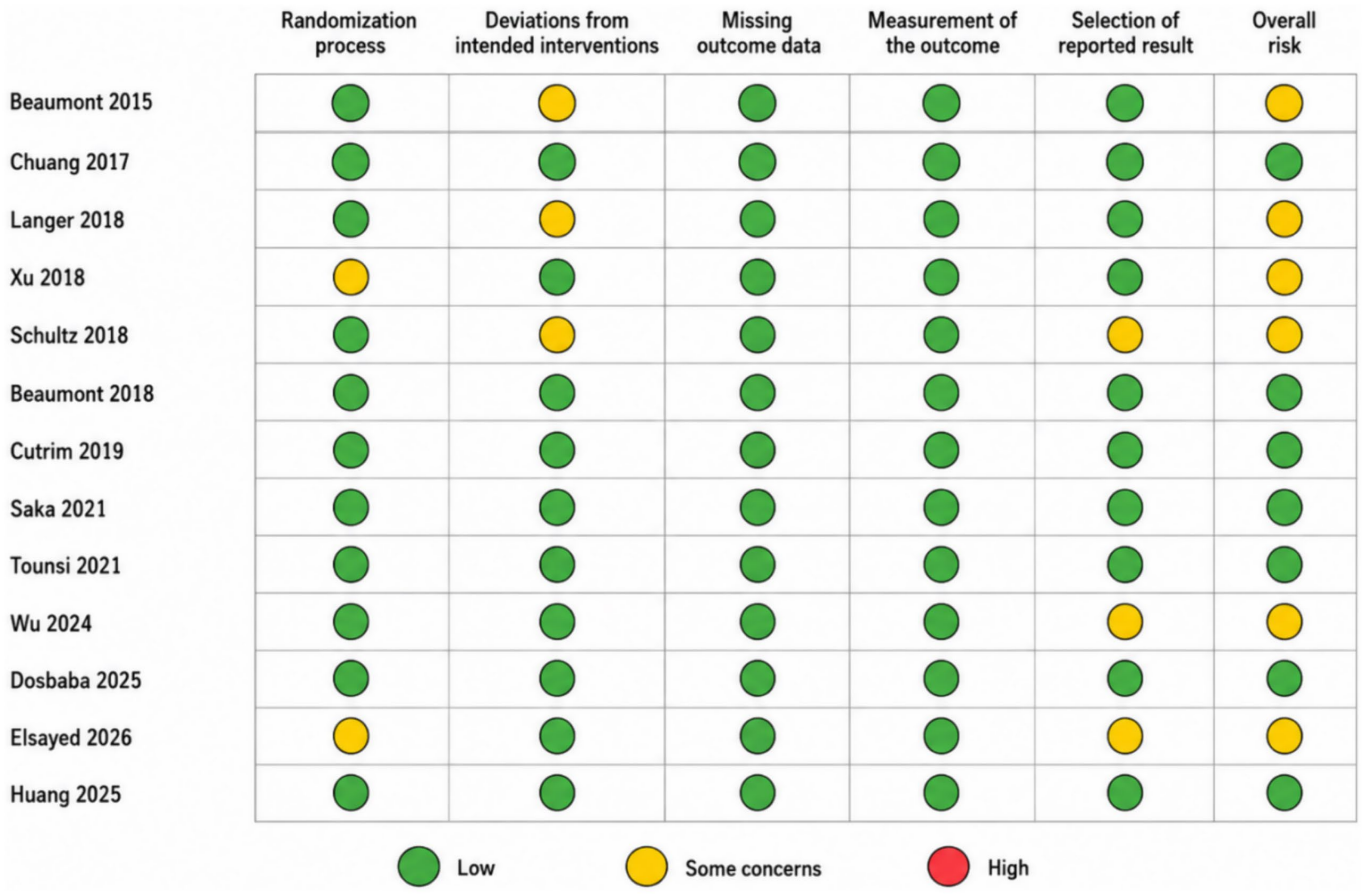
Traffic-light plot of study-level risk of bias judgments (RoB).

The risk-of-bias assessment according to RoB 2.0 is summarized in Supplementary Table S1. Among the 13 included RCTs, 6 were judged as “low risk” overall, 5 had “some concerns,” and 2 were rated “high risk.” The domains most frequently flagged were deviations from intended interventions (4 studies with “some concerns”) and selection of the reported result (3 studies with “high risk”). A visual summary of study-level judgments is presented in Figure 2 (traffic-light plot). These judgments provide a more granular view of methodological quality than previously reported using RoB 1.0 and allow readers to interpret the pooled results in light of study-specific biases.

The detailed GRADE assessment for each main outcome is presented in Supplementary Table S2.

### 3.3 PImax outcome meta-analysis results

**Figure 3:**
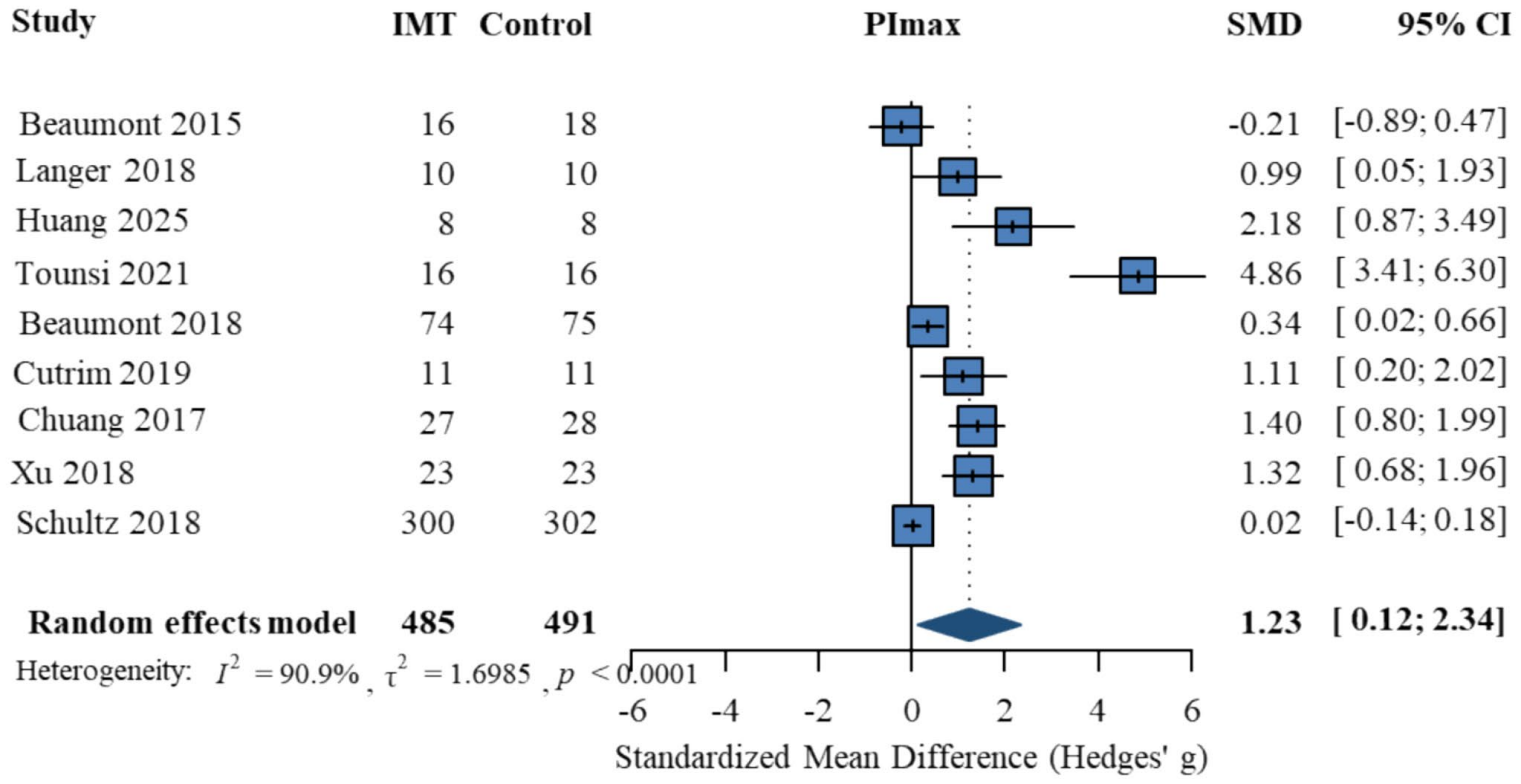
Overall forest plot for PImax.

Nine studies reported PImax outcomes, with a total of 976 participants (485 in IMT group, 491 in control group). Because the original reports provided PImax data in two different formats—change scores and post-treatment values—we opted for a random-effects model to pool Hedges’ g (SMD). According to the overall analysis, IMT led to a significant increase in PImax in the COPD population, with a summary effect size of 1.23 (95% CI: 0.12–2.34, *p* = 0.034). However, heterogeneity across studies was high (*I*^2^ = 90.9%, τ^2^ = 1.70, Q test *p* < 0.0001). Looking at individual studies, most favored IMT for PImax, but effect sizes varied considerably. Tounsi 2021 showed the largest effect (SMD = 4.86, 95%CI 3.41–6.30), whereas Schultz 2018 showed no clear between-group difference (SMD = 0.02, 95%CI −0.14–0.18); Beaumont 2015 showed an effect direction favoring the control group, but the difference was not statistically significant (SMD = −0.21, 95%CI −0.89–0.47) (Figure 3).

**Figure 4:**
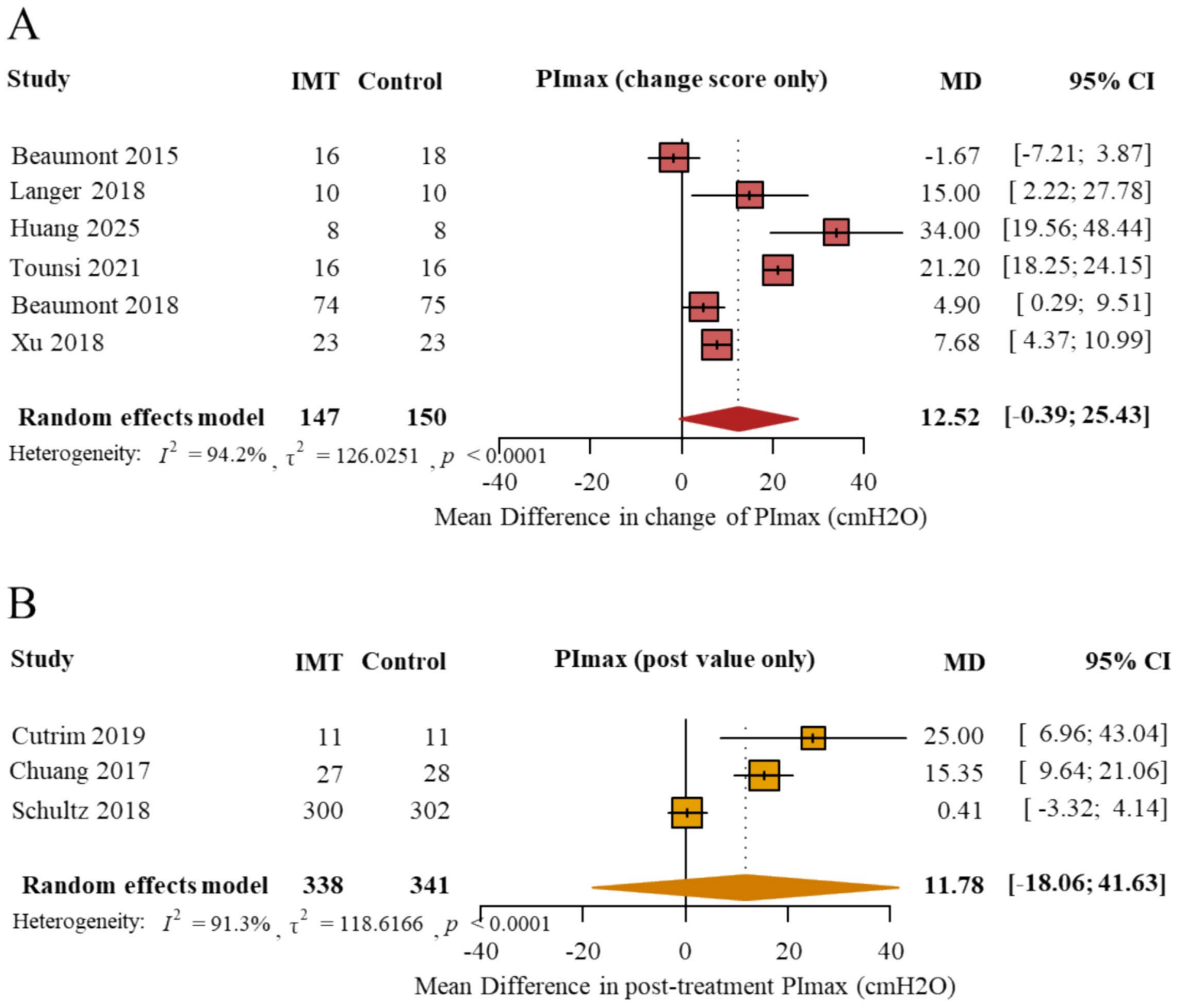
Effects of inspiratory muscle training on PImax in COPD patients. **(A)** Change-score-based meta-analysis of PImax comparing IMT versus control. **(B)** Post-intervention value-based meta-analysis of PImax comparing IMT versus control. Results are presented as standardized mean differences (Hedges’ g) with 95% confidence intervals using a random-effects model.

When only change-score studies were included, a supplementary analysis using MD showed that the IMT group had a 12.52 cmH₂O greater improvement in PImax compared with controls (95%CI −0.39–25.43, *p* = 0.055), with high heterogeneity (*I*^2^ = 94.2%). When only post-treatment value studies were included, the pooled MD was 11.78 cmH_2_O (95%CI −18.06–41.63, *p* = 0.231), also with high heterogeneity (*I*^2^ = 91.3%). These supplementary analyses were directionally consistent with the main analysis, generally supporting the improvement in PImax with IMT, but the effect estimates were uncertain due to high heterogeneity (Figure 4).

When stratified by comparator type, the background-matched add-on subgroup (3 studies) had a pooled SMD = 1.60 (95%CI −5.23–8.43, *p* = 0.420); the sham-based subgroup (4 studies) had SMD = 1.00 (95%CI −0.41–2.41, *p* = 0.110); the usual/no intervention subgroup (2 studies) had SMD = 1.31 (95%CI −0.34–2.97, *p* = 0.063). Heterogeneity within subgroups was high or sample sizes were limited. Meta-regression suggested that comparator type did not significantly explain between-study heterogeneity (QM = 0.113, *p* = 0.945) (Table 3).

**Table 3:** Subgroup analysis results by comparator type for PImax.

Comparator subgroup	*k*	Effect	LCI	UCI	*p* value	*I* ^2^	τ^2^
Background-matched add-on	3	1.597	−5.234	8.429	0.42	0.95	7.177
Sham-based	4	0.996	−0.413	2.405	0.11	0.892	0.639
Usual/no intervention	2	1.314	−0.341	2.969	0.063	0	0

**Figure 5:**
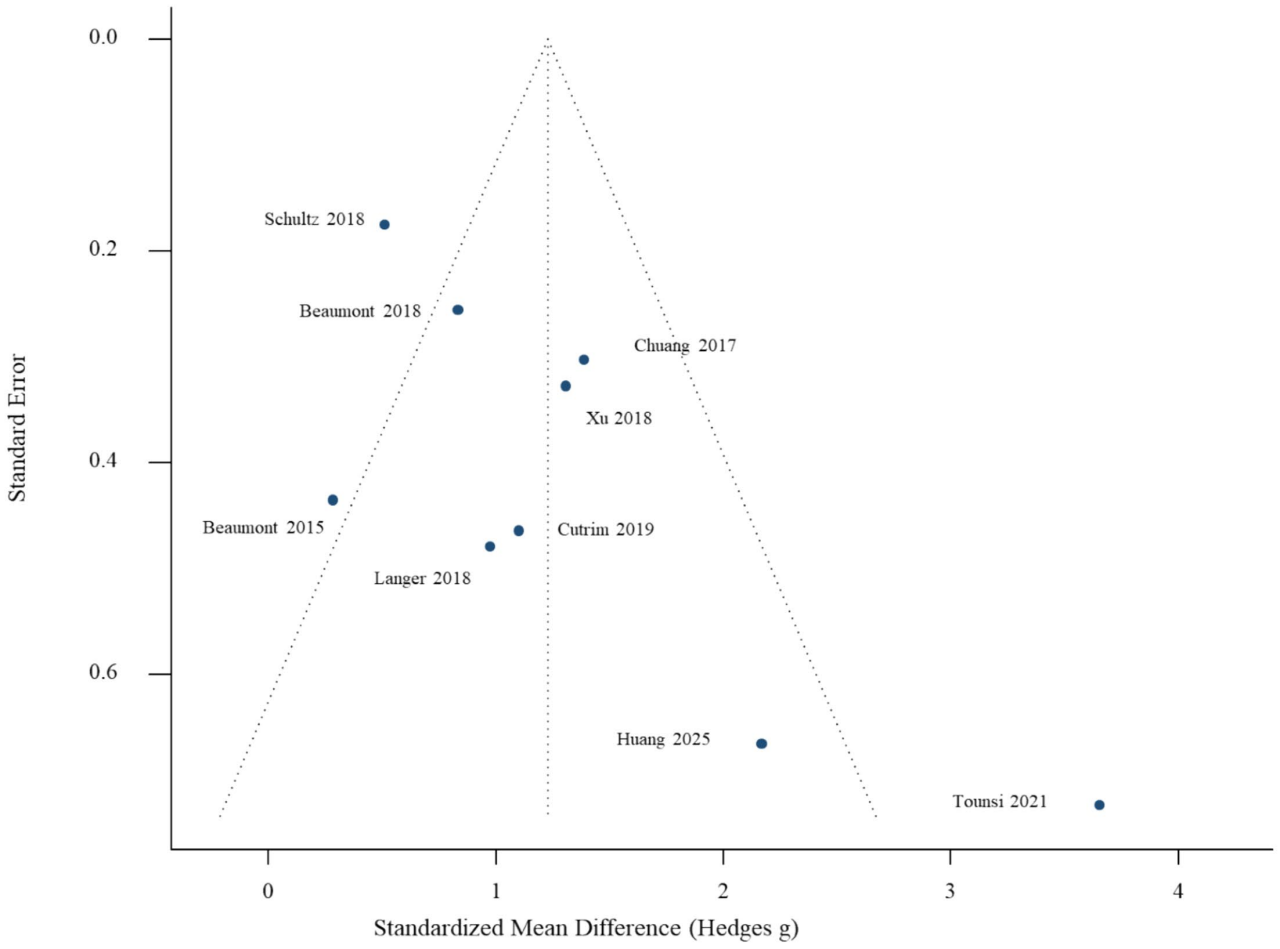
Funnel plot of PImax.

Sensitivity analyses showed that the results were generally robust. The main analysis for PImax showed consistent improvement with IMT. Full leave-one-out sensitivity analyses and detailed influence assessments are provided in Supplementary Figure S1 to support the robustness of the pooled effect. Due to the small number of included studies and high heterogeneity, publication bias assessment should be interpreted cautiously. We mainly used a funnel plot for descriptive judgment and did not draw definitive conclusions about publication bias (Figure 5).

### 3.4 6MWD outcome meta-analysis results

Eleven studies reported 6MWD outcomes, with a total of 1,050 participants (522 in IMT group, 528 in control group). Because the included studies contained both change-score and post-treatment value data forms, the main analysis used a random-effects model to pool SMD (Hedges’ g). The overall analysis showed that IMT was significantly associated with improved 6MWD, with a pooled SMD = 0.43 (95%CI 0.09–0.77, *p* = 0.018). In MD terms, the pooled improvement was 39.3 m (95% CI −3.2–81.8 m), exceeding the commonly accepted MCID for 6MWD in COPD patients (~25–30 m), suggesting the change is likely to be clinically meaningful despite statistical uncertainty. There was moderate to high heterogeneity across studies (*I*^2^ = 70.6%, τ^2^ = 0.169, *p* = 0.0002).

**Figure 6:**
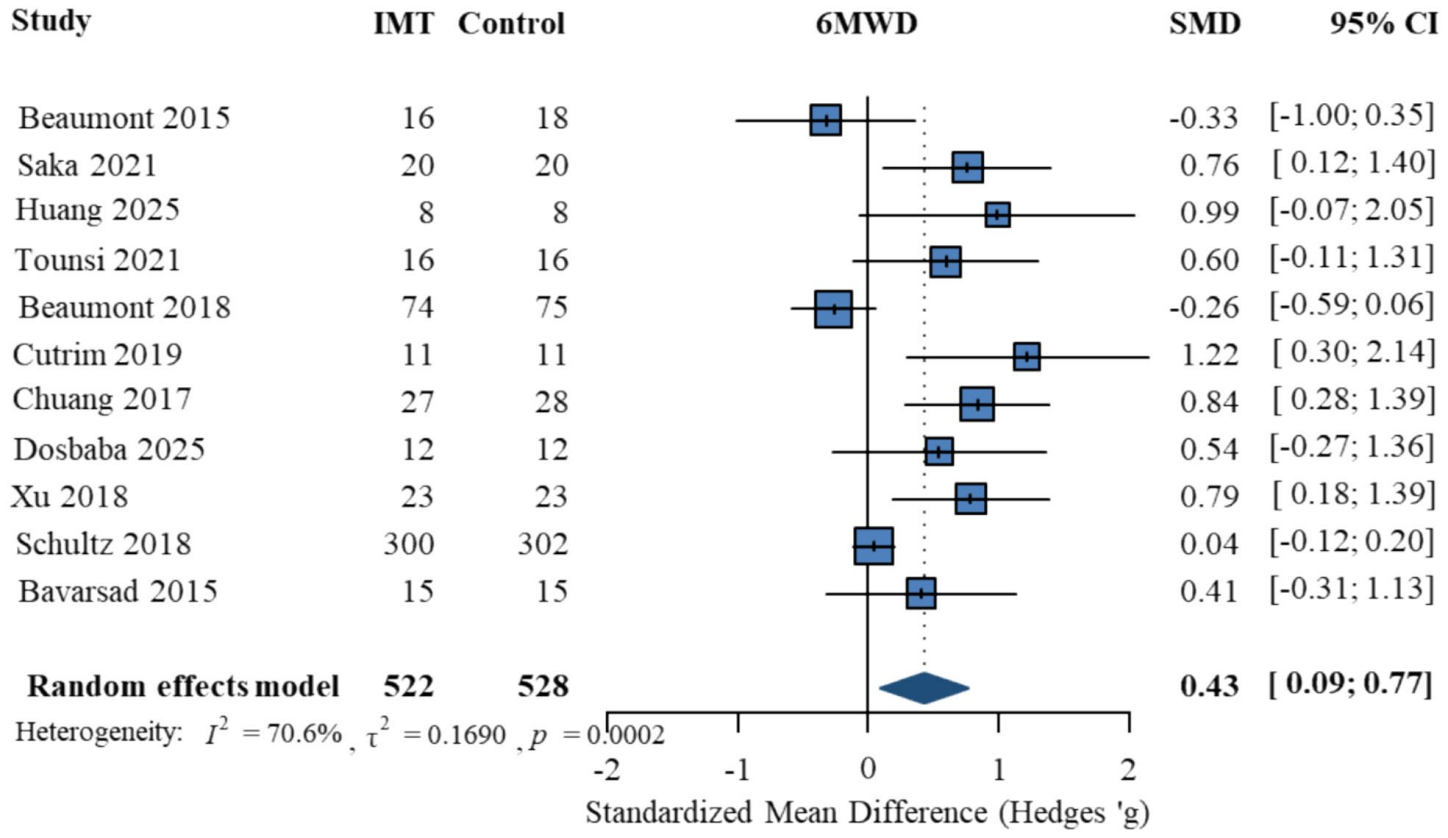
Overall forest plot for 6MWD.

Looking at individual studies, most had effect directions favoring IMT, but the magnitudes varied. Cutrim 2019 showed the largest effect (SMD = 1.22, 95%CI 0.30–2.14), whereas Schultz 2018 showed a very small between-group difference (SMD = 0.04, 95%CI −0.12–0.20). Beaumont 2015 and Beaumont 2018 showed effect directions favoring the control group, but the differences were not statistically significant (Figure 6).

**Figure 7:**
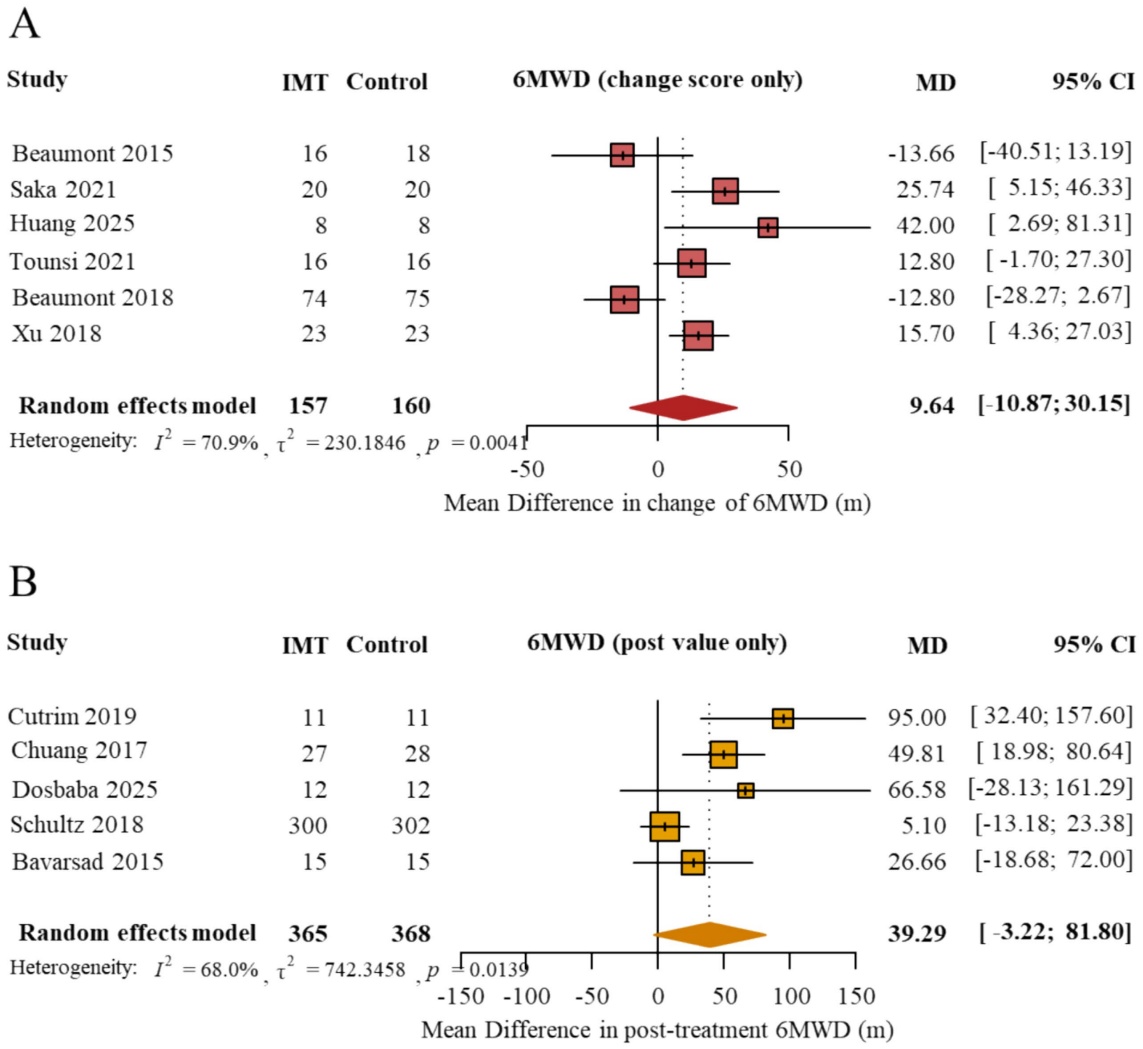
Effects of inspiratory muscle training on 6-minute walk distance (6MWD) in COPD patients. **(A)** Change-score-based meta-analysis of 6MWD comparing IMT versus control. **(B)** Post-intervention value-based meta-analysis of 6MWD comparing IMT versus control. Effect estimates are expressed as standardized mean differences (Hedges’g) with 95% confidence intervals.

When only change-score studies were included, a supplementary analysis using MD showed that the IMT group had a 9.64 m greater improvement in 6MWD compared with controls (95%CI −10.87–30.15 m, *p* = 0.281), which is below the MCID threshold of ~25–30 m. When only post-treatment value studies were included, the pooled MD was 39.29 m (95%CI −3.22–81.80 m, *p* = 0.062), exceeding the MCID, indicating a potentially meaningful improvement in walking capacity. Overall, the MD analyses were directionally consistent with the main analysis, suggesting that IMT may improve 6MWD, but effect estimates were uncertain due to wide confidence intervals and heterogeneity (Figure 7).

When analyses were stratified according to comparator type, the background-matched add-on subgroup (three studies) yielded a pooled SMD of −0.05 (95% CI: −1.25–1.15, *p* = 0.875). In the sham-based subgroup (five studies), the pooled SMD was 0.51 (95% CI: 0.02–1.01, *p* = 0.046), while the usual-care or no-intervention subgroup (three studies) produced a pooled SMD of 0.78 (95% CI: −0.07–1.63, *p* = 0.059). Statistical significance was achieved only in the sham-controlled subgroup. Meta-regression analysis revealed that comparator type accounted for approximately 37.8% of the observed heterogeneity, with a result approaching statistical significance (QM = 5.57, *p* = 0.062). Further modeling indicated that, relative to the reference category, studies employing usual care or no intervention showed a larger effect size (regression coefficient = 0.848, 95% CI: 0.113–1.583, *p* = 0.024). Meanwhile, sham-based controls exhibited a borderline significant trend (regression coefficient = 0.557, 95% CI: −0.069–1.183, *p* = 0.081) (Table 4).

**Table 4:** Subgroup analysis results by comparator type for 6MWD.

Comparator subgroup	*k*	Effect	LCI	UCI	*p* value	*I* ^2^	τ^2^
Background-matched add-on	3	−0.05	−1.246	1.147	0.875	0.598	0.13
Sham-based	5	0.511	0.016	1.006	0.046	0.685	0.122
Usual/no intervention	3	0.779	−0.074	1.632	0.059	0	0

The pooled 6MWD effect remained consistent across studies, with supplementary leave-one-out and publication bias analyses provided in Supplementary Figures S2, S3. The main manuscript focuses on the primary effect estimates (SMD = 0.43, 95% CI 0.09–0.77) to highlight clinically relevant improvements, while detailed sensitivity and bias plots are moved to the supplement for clarity.

### 3.5 Dyspnea-related scale meta-analysis results

**Figure 8:**
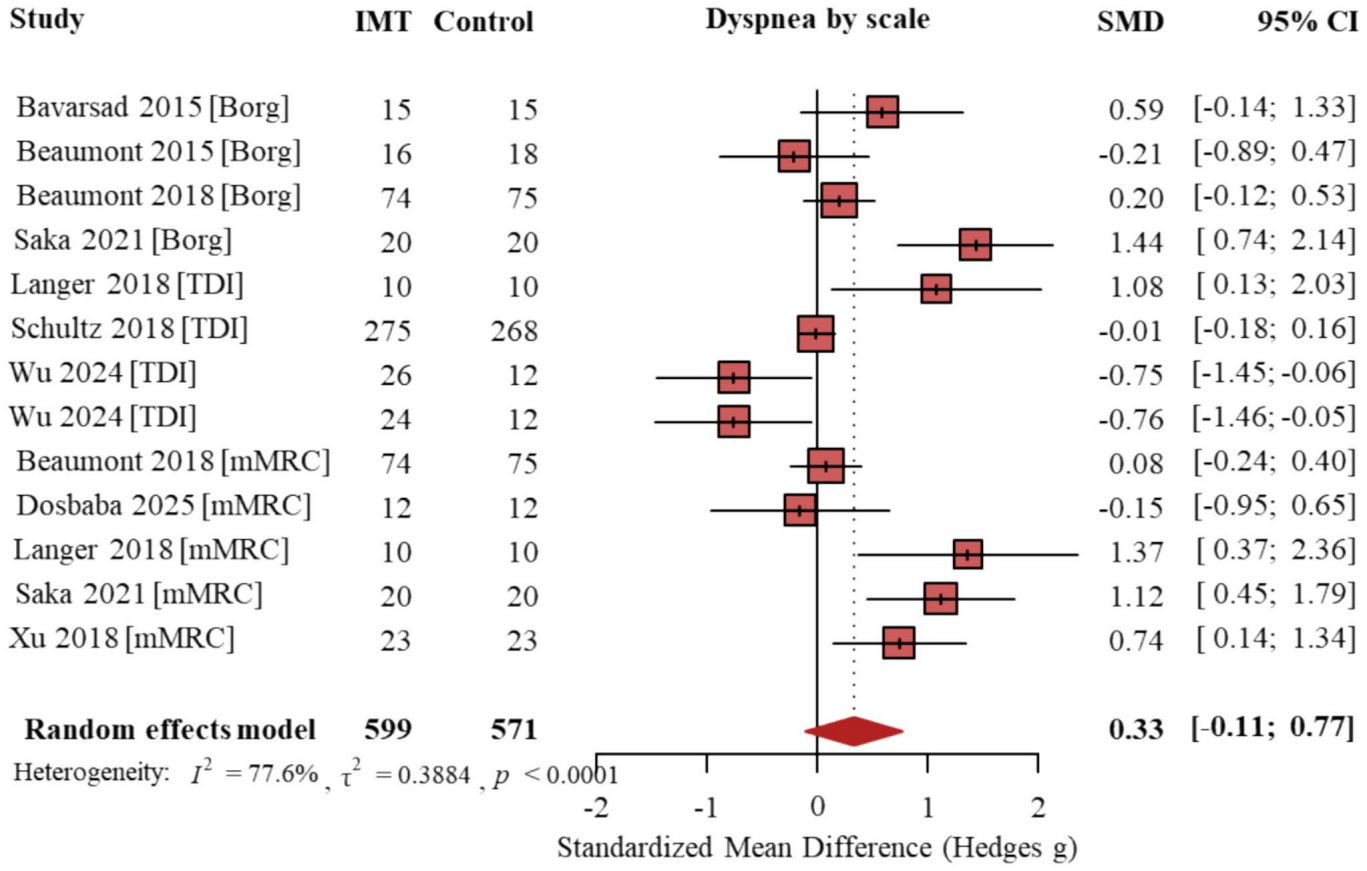
Overall forest plot for Dyspnea.

This systematic review incorporated 13 studies with a total of 1,170 participants (599 in the IMT group and 571 in the control group) that evaluated dyspnea using scales such as Borg, mMRC, and TDI. Before pooling, all dyspnea outcomes were harmonized so that positive SMD values indicated improvement; for Borg and mMRC, where lower scores indicate less dyspnea, effect directions were reversed when necessary, whereas TDI was retained in its original direction because higher values indicate clinical improvement. The primary analysis employed a random-effects model to pool standardized mean differences (SMD, Hedges’ g). The overall pooled result indicated a favorable trend of IMT on dyspnea that did not reach statistical significance, with a pooled SMD of 0.33 (95% CI: −0.11–0.77, *p* = 0.129) and substantial heterogeneity (*I*^2^ = 77.6%, τ^2^ = 0.388, *p* < 0.0001). Effect sizes varied widely across studies: some reported clear improvements (e.g., Saka 2021, SMD = 1.44, 95% CI: 0.74–2.14), whereas others showed weak or near-null effects (e.g., Beaumont 2015, SMD = −0.21, 95% CI: −0.89–0.47) (Figure 8).

**Figure 9:**
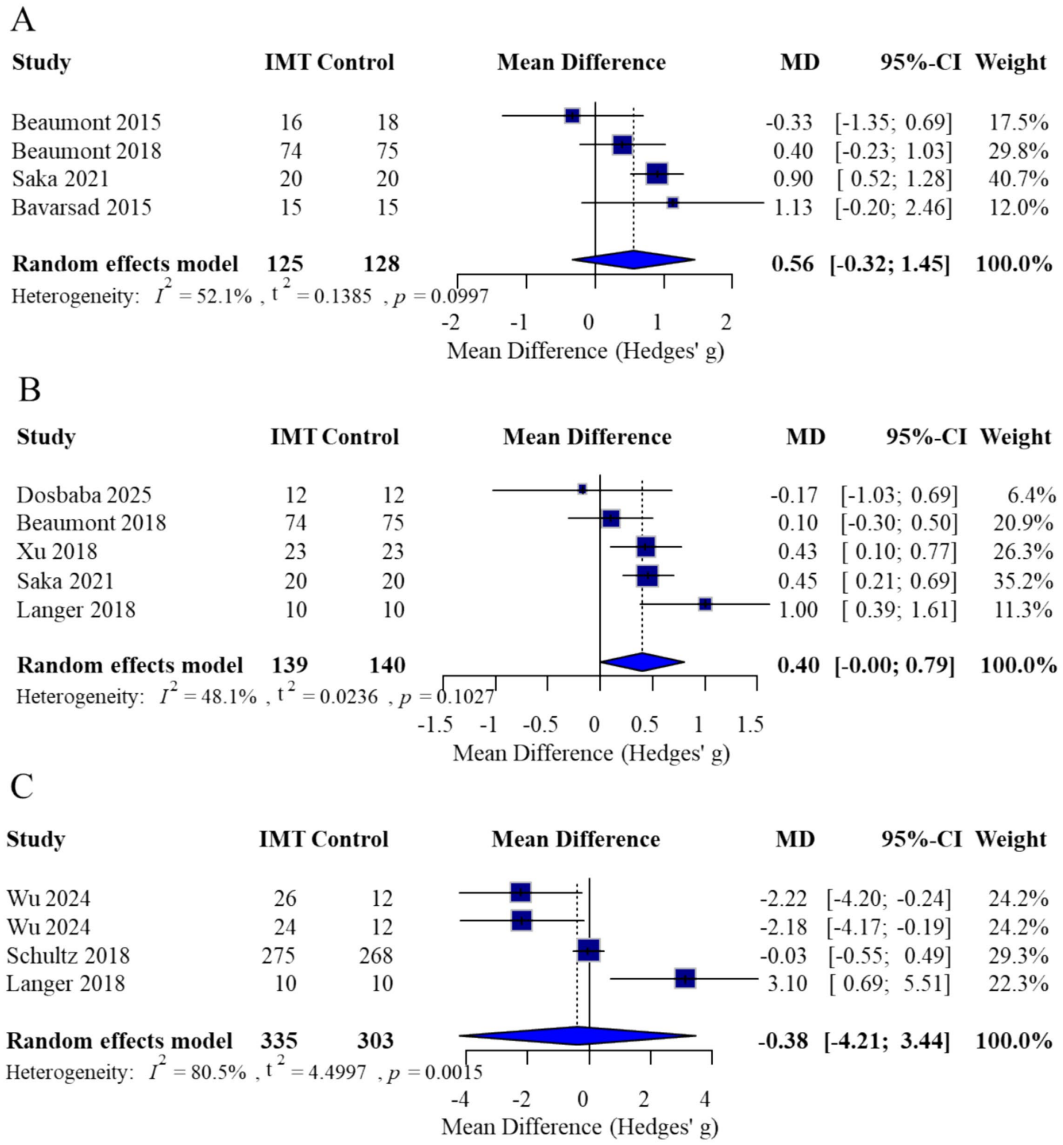
Subgroup meta-analysis of dyspnea outcomes according to measurement scale. **(A)** Studies using Borg scale for dyspnea assessment. **(B)** Studies using mMRC scale for dyspnea assessment. **(C)** Studies using Transition Dyspnea Index (TDI) for dyspnea assessment. All effect sizes are standardized mean differences (Hedges’g) with 95% confidence intervals using a random-effects model.

When further stratified by scale type, both the Borg (four studies) and mMRC (five studies) subgroups showed trends toward improvement after direction harmonization, with pooled effects of 0.56 (95% CI: −0.32–1.45; *I*^2^ = 52.1%) and 0.40 (95% CI: −0.00–0.79; *I*^2^ = 48.1%), respectively. The TDI subgroup (four studies) showed a weaker and non-significant effect (SMD = −0.38, 95% CI: −4.21–3.44; *I*^2^ = 80.5%). These scale-specific analyses should be interpreted cautiously because the scales capture different dyspnea constructs, including daily-life dyspnea, exertional dyspnea, and transition-based clinical change. Scale type did not significantly explain the between-study variation (Figure 9).

**Figure 10:**
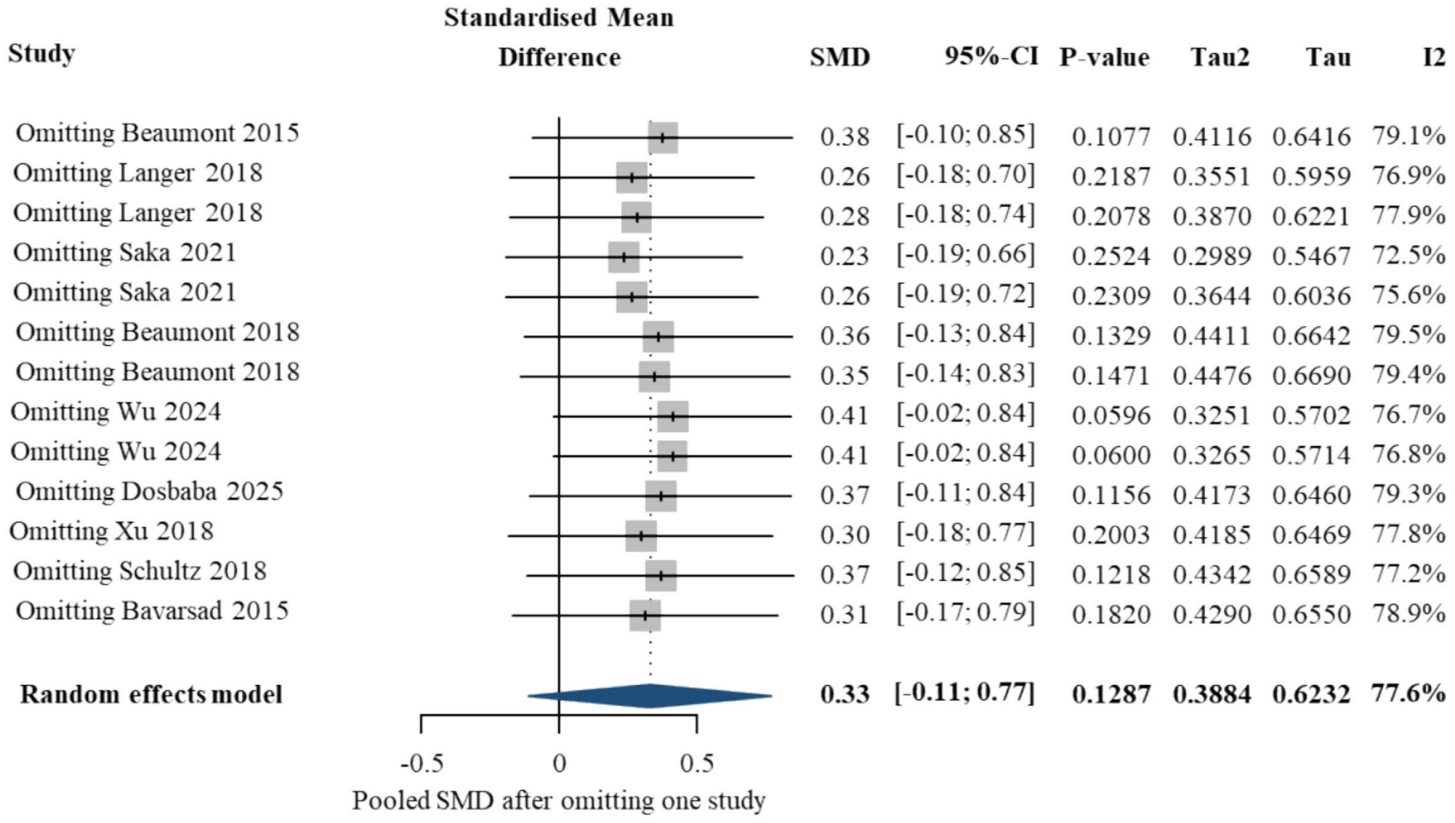
Leave-one-out analysis of Dyspnea.

**Figure 11:**
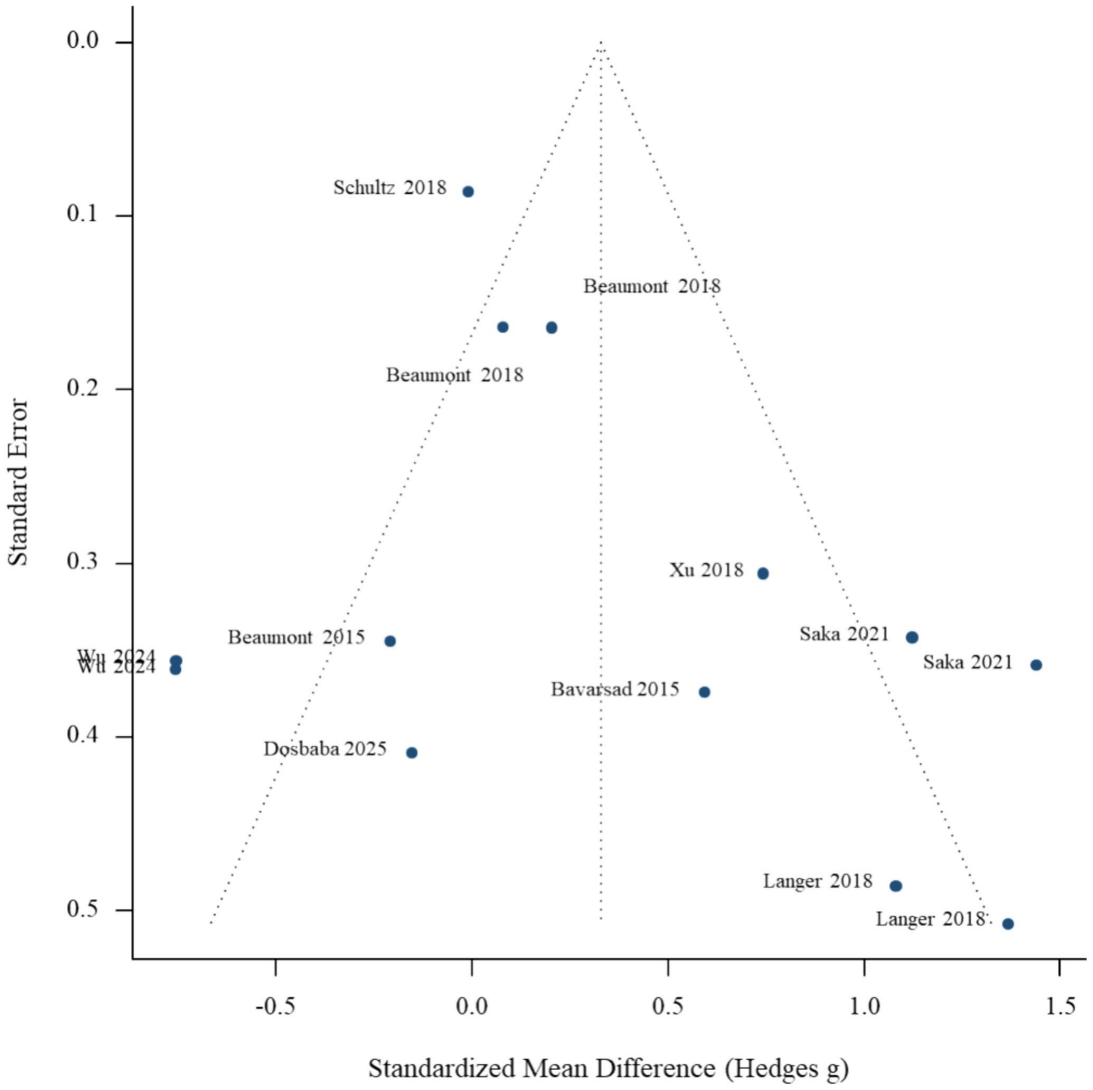
Funnel plot of Dyspnea.

Sensitivity analyses confirmed the robustness of the results. Leave-one-out analyses showed that after removing individual studies one by one, the pooled SMD ranged from 0.26 to 0.41, and none of the iterations reached statistical significance, indicating stable findings (Figure 10). The outcome of Egger’s test did not reach statistical significance for small-study effects (*p* = 0.170), as shown in Figure 11.

### 3.6 Core lung function analysis results

**Figure 12:**
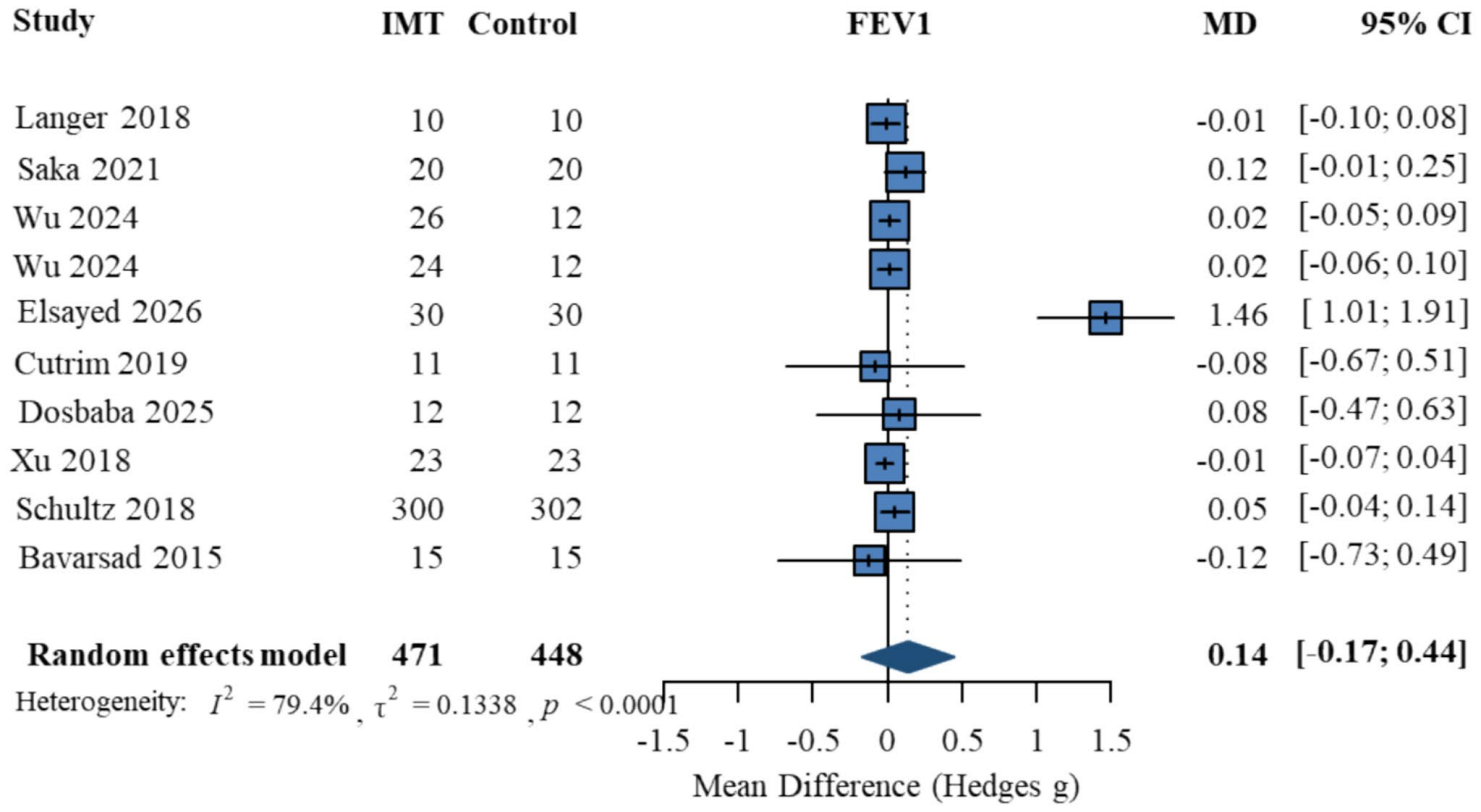
Overall forest plot for FEV₁.

The FEV₁ analysis incorporated ten comparisons, representing a combined sample of 919 individuals (471 assigned to IMT and 448 to the control condition). Although % predicted FEV_1_ values were used to verify COPD severity and eligibility when reported, FEV_1_ values in liters were extracted for quantitative synthesis because this was the most consistently available unit across the included trials. Applying a random-effects model, the pooled mean difference was 0.14 L (95% CI: −0.17–0.44, *p* = 0.190), indicating that IMT did not produce a statistically significant improvement in FEV_1_. Across the included studies, heterogeneity was substantial (*I*^2^ = 79.4%, τ^2^ = 0.134, Q test *p* < 0.0001) (Figure 12).

**Figure 13:**
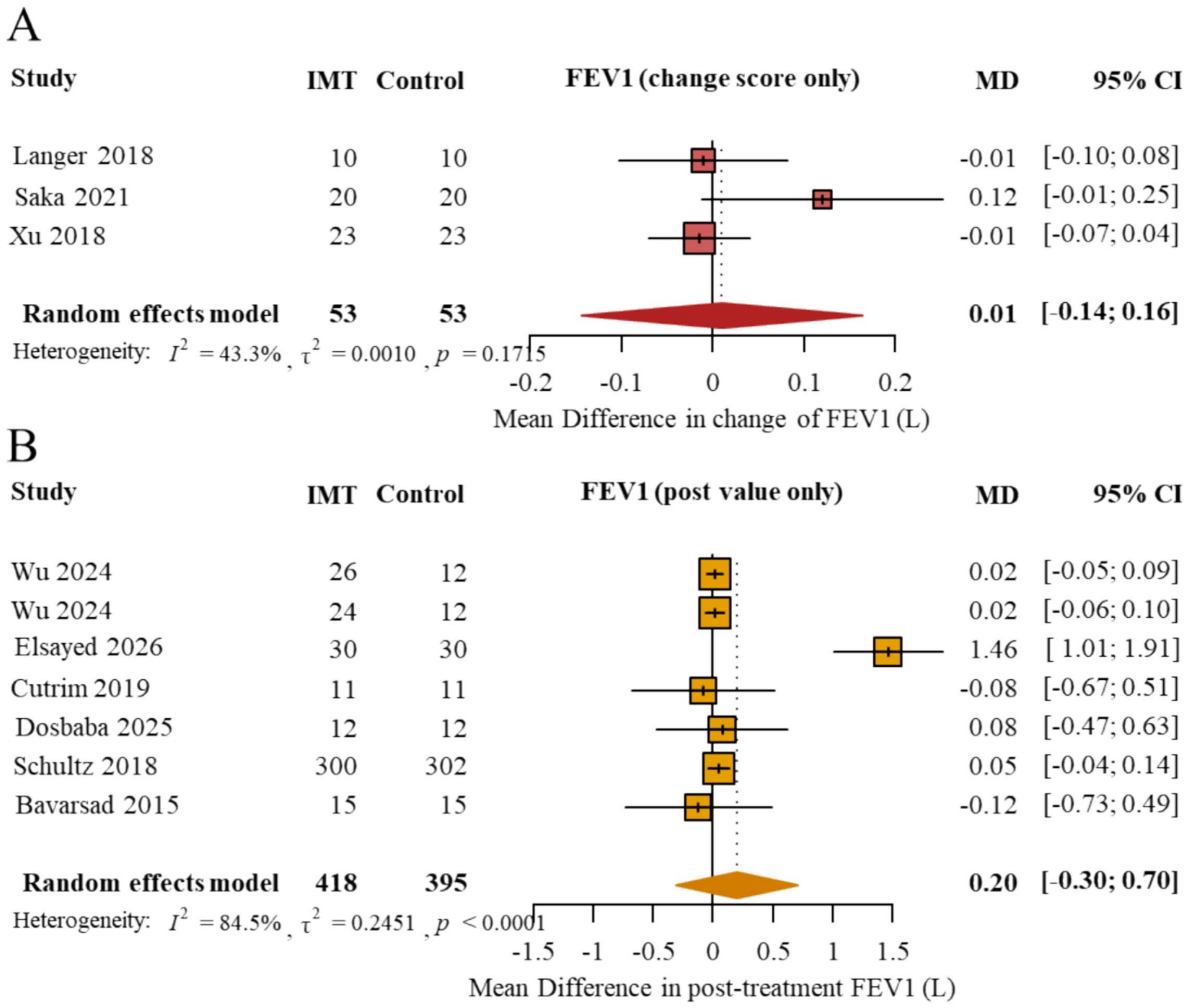
Effects of inspiratory muscle training on forced expiratory volume in 1 second (FEV_1_) in COPD patients. **(A)** Change-score-based meta-analysis of FEV_1_ comparing IMT versus control. **(B)** Post-intervention value-based meta-analysis of FEV_1_ comparing IMT versus control. Results are presented as mean differences with 95% confidence intervals using a random-effects model.

Restricting the analysis to change-score studies revealed no significant effect of IMT on FEV₁ change (MD = 0.01 L, 95% CI: −0.14–0.16, *p* = 0.805; *I*^2^ = 43.3%). Likewise, when only post-treatment values were examined, the summary estimate remained non-significant (MD = 0.20 L, 95% CI: −0.30–0.70, *p* = 0.369; *I*^2^ = 84.5%) (Figure 13).

After grouping studies by comparator type, the sham-controlled subset gave a pooled MD of 0.09 (95% CI: −0.08–0.27, *p* = 0.216; *I*^2^ = 0%), while the usual-care or no-intervention subset yielded a pooled MD of 0.05 (95% CI: −0.24–0.34, *p* = 0.620; *I*^2^ = 0%). One background-matched add-on trial (Elsayed 2026) showed a markedly positive effect (SMD = 1.61, 95% CI: 1.02–2.19). Meta-regression pointed to comparator type as a principal driver of between-study heterogeneity (*p* < 0.0001) (Table 5).

**Table 5:** Subgroup analysis results by comparator type for FEV_1_.

Comparator subgroup	*k*	Effect	Lower_CI	Upper_CI	*p*_value	*I* ^2^	τ^2^
Sham-based	5	0.093	−0.083	0.269	0.216	0	0
Usual/no intervention	4	0.05	−0.237	0.336	0.62	0	0

**Figure 14:**
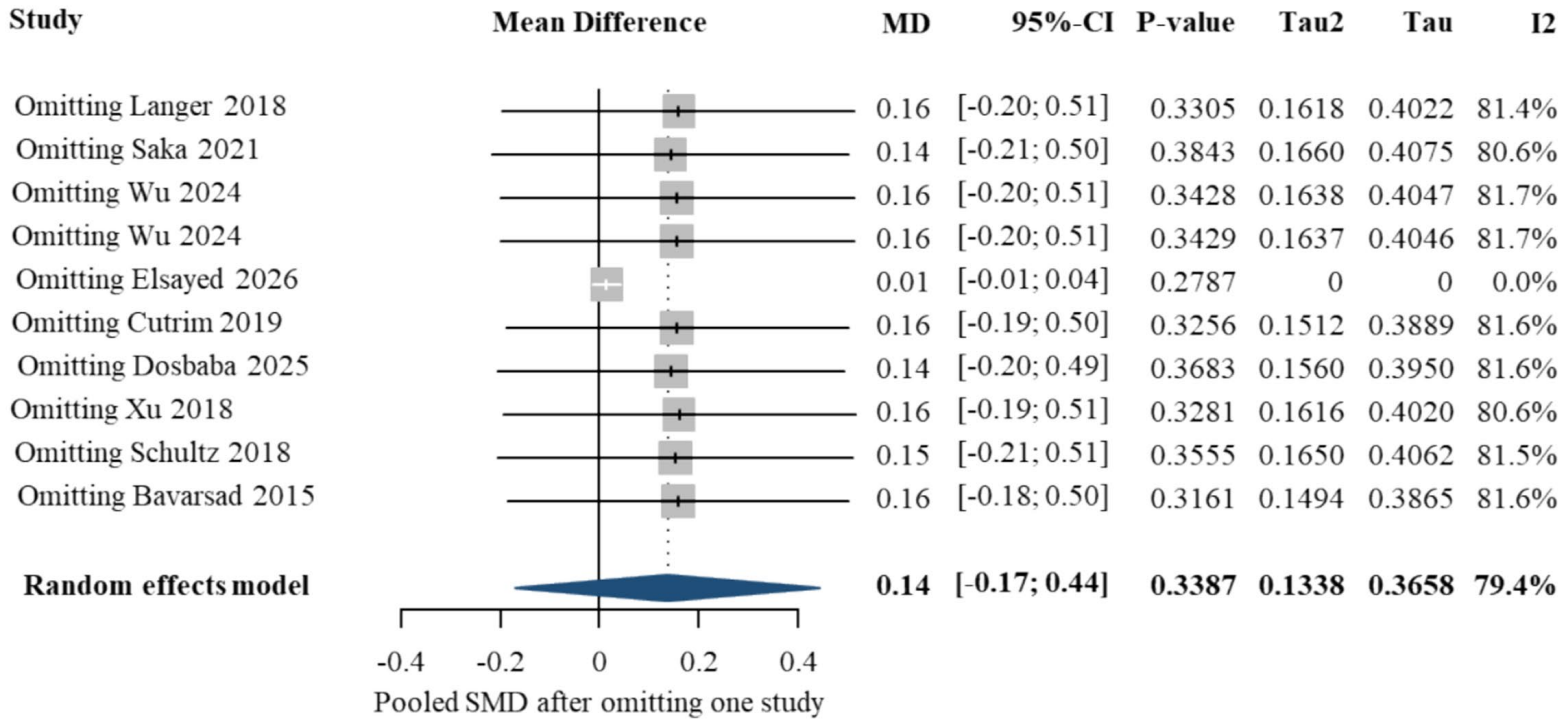
Leave-one-out analysis of FEV₁.

**Figure 15:**
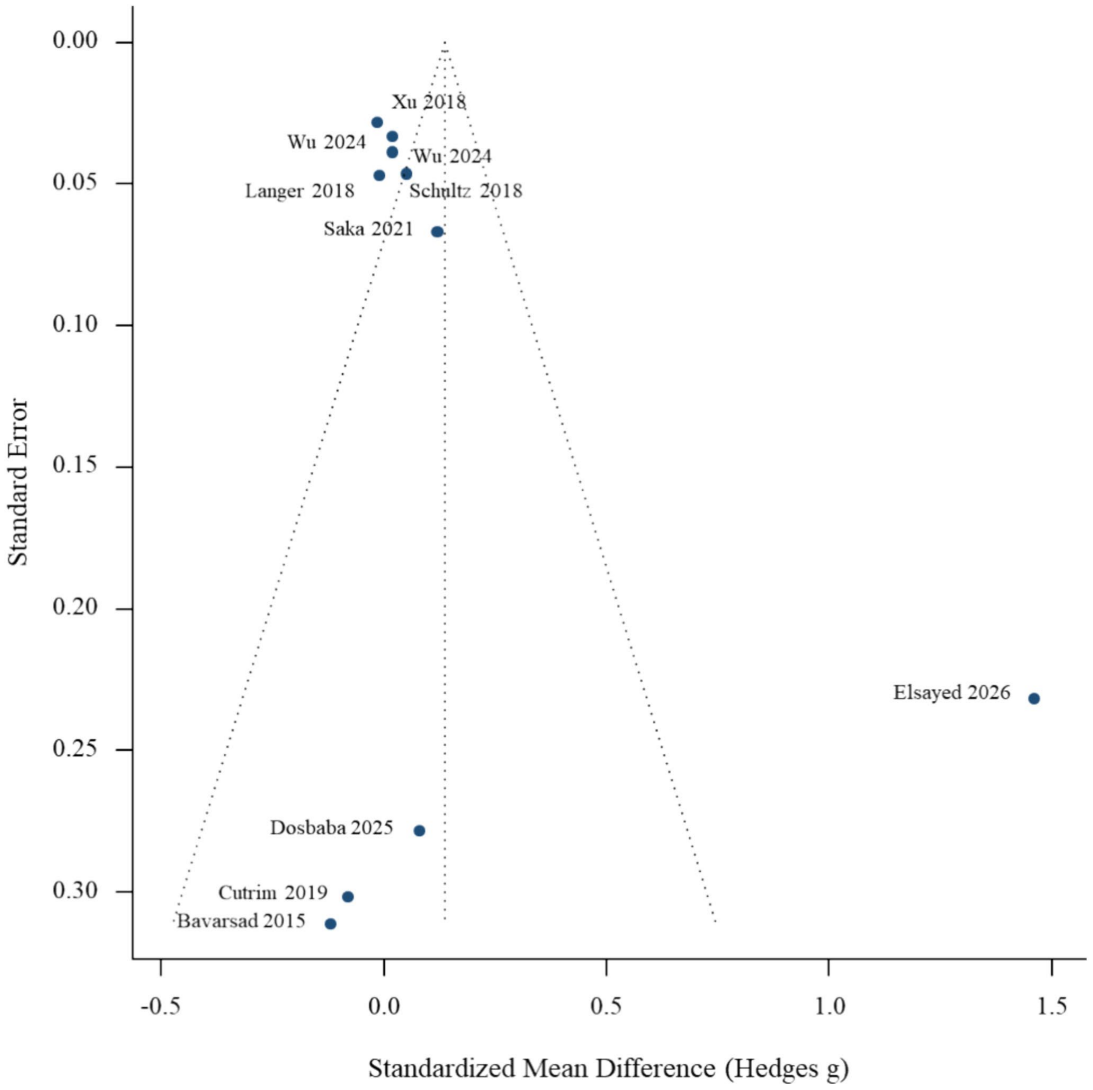
Funnel plot of FEV_1_.

Sensitivity analysis, after removal of the largest study (Schultz 2018), still found no significant effect (MD = 0.15, 95% CI: −0.21–0.51, *p* = 0.3555; *I*^2^ = 81.5%). Leave-one-out analysis suggested that the overall result was fairly robust; however, excluding Elsayed 2026 caused the pooled effect to drop to MD = 0.01 (95% CI: −0.01–0.04, *p* = 0.2787) and heterogeneity to fall from 79.4 to 0%, indicating that this study contributed notably to both the overall estimate and the heterogeneity (Figure 14). The funnel plot exhibited no clear asymmetry, and Egger’s test gave no evidence of significant small-study effects (*p* = 0.580) (Figure 15).

### 3.7 Sensitivity analysis excluding post-AECOPD rehabilitation-phase participants

After exclusion of Huang 2025 (25), the pooled effect on PImax remained favorable and statistically significant (SMD = 1.02, 95% CI: 0.42–1.62, *p* = 0.001; *I*^2^ = 91.3%). The effect on 6MWD also remained significant, although the pooled estimate was slightly reduced (SMD = 0.39, 95% CI: 0.08–0.69, *p* = 0.012; *I*^2^ = 71.6%). Huang 2025 did not contribute data to the dyspnea or FEV₁ analyses; therefore, the pooled estimates for these outcomes remained unchanged (dyspnea: SMD = 0.33, 95% CI: −0.11–0.77, *p* = 0.129; *I*^2^ = 77.6%; FEV_1_: MD = 0.14 L, 95% CI: −0.17–0.44, *p* = 0.190; *I*^2^ = 79.4%). These findings suggest that inclusion of the post-AECOPD rehabilitation-phase study did not materially alter the overall conclusions, although the presence of only one post-AECOPD study precluded a reliable subgroup comparison by disease phase. The results are summarized in Supplementary Table S3.

## 4 Discussion

Based on 13 randomized controlled trials, this systematic review and meta-analysis aimed to determine whether IMT improves clinical outcomes in patients with COPD, including both stable-phase and post-AECOPD rehabilitation-phase participants. The overall analysis indicated that IMT significantly improved patients’ PImax and exercise tolerance (6MWD), whereas improvements in FEV₁ and dyspnea scales did not reach statistical significance. In an exploratory comparator-based subgroup analysis, we assessed the effect of different comparator types on the pooled estimates. The findings suggested that the apparent treatment effect of IMT may vary according to the control condition, particularly for 6MWD, but these results should be interpreted cautiously because the protocol was registered retrospectively and the number of studies in each subgroup was limited.

The significant improvement in PImax with IMT confirms its effectiveness as a targeted therapy to reverse inspiratory muscle weakness in patients. Compared with analyses that pooled different comparator conditions together, our exploratory findings suggest that the incremental benefit of IMT may be influenced by comparator type. Specifically, the improvement in 6MWD with IMT was particularly evident in the sham-controlled subgroup, whereas its additional benefit was negligible in the “background-matched add-on” setting (i.e., adding IMT to standard PR versus PR alone). This finding is highly consistent with the “ceiling effect” hypothesis proposed in recent pulmonary rehabilitation research: when patients already receive structured PR, their cardiopulmonary and peripheral skeletal muscle systems may have reached a physiological compensatory limit, at which point a single targeted intervention (such as IMT) is unlikely to yield additional clinical benefit (26, 27). Because high-standard whole-body PR already imposes sufficient load on the cardiopulmonary and peripheral skeletal muscle systems, effectively approaching the patient’s physiological compensatory limit, simply adding a single inspiratory resistance intervention can hardly release significant spillover value in overall exercise endurance (28). However, for COPD patients who are elderly, severely deconditioned, or have comorbid cardiovascular disease, whole-body exercise training may carry a risk of acute events; in such cases, IMT alone, as a low-load, non-exercise-dependent intervention, can safely improve inspiratory muscle function and translate into improved walking ability (29). Leave-one-out sensitivity analyses did not materially change the direction of the main findings, although the results should still be interpreted cautiously given the high heterogeneity and limited number of studies in several subgroup analyses. The overall certainty of the evidence is influenced by the risk-of-bias profile of included studies. While most RCTs were at low or moderate risk, two studies had high risk due to selective reporting or deviations from intended interventions. Using RoB 2.0 provides outcome-specific judgments, indicating that the observed heterogeneity in PImax and 6MWD may partly reflect differences in trial conduct and reporting. It is encouraged to interpret pooled effect sizes in light of these study-level biases, particularly for outcomes with high heterogeneity and those influenced by subjective measurement scales, such as dyspnea. Multiple sources contributed to the observed heterogeneity across included studies. First, the distribution of COPD severity varied widely, from GOLD I to IV, which could influence baseline exercise tolerance and respiratory muscle strength. Second, participants’ baseline inspiratory muscle weakness differed, with some studies enrolling patients with markedly reduced PImax, leading to larger relative gains, whereas others included patients with near-normal inspiratory strength. Third, training intensity varied from 30 to 80% of PImax across studies, which likely affected the magnitude of improvements in PImax and 6MWD. Fourth, supervision varied from fully supervised, clinic-based training to unsupervised home-based programs, influencing adherence and intervention fidelity. Collectively, these factors contributed to substantial between-study heterogeneity (*I*^2^ ranging from 70 to 90% for primary outcomes). This high heterogeneity limits the certainty of pooled effect estimates and reduces generalizability, indicating that the observed effect sizes should be interpreted cautiously, and individualized adjustments may be necessary when applying IMT in clinical practice.

The lack of significant improvement in dyspnea perception and the lack of change in lung function can be explained from multiple physiological and psychological pathways. First, there are substantial psychometric and contextual differences among dyspnea scales. The mMRC scale mainly reflects functional limitation related to daily-life breathlessness, Borg scores commonly capture exertional dyspnea during or after exercise testing, and TDI reflects transition-based clinical change. Although we harmonized all scales so that positive SMD values represented improvement and avoided double counting by selecting or combining multiple dyspnea outcomes at the study level, residual heterogeneity in scale constructs and MCID thresholds may still have diluted the pooled effect. More importantly, dyspnea is a multidimensional syndrome with strong subjective characteristics. Simply reducing the mechanical work of breathing without combining effective patient cognitive and behavioral guidance to reduce the emotional threshold panic related to “suffocation and shortness of breath” is unlikely to achieve substantial breakthroughs in subjective limitation in clinical practice (30–32). Furthermore, due to the core pathological features of COPD, such as irreversible small airway inflammatory remodeling and extensive destruction of elastic tissue (33), IMT, as a non-invasive peripheral intervention targeting the ventilatory pump, can effectively improve the contractile strength (work efficiency) of the respiratory muscles, but its mechanism does not involve direct modification of lung parenchyma or airway pathology, nor can it reverse the inherent ventilation impairment (e.g., improvement in FEV₁) or maintain distal airway patency from an anatomical perspective (6, 9). This is the objective physiological basis for why no statistically significant improvement in FEV_1_ or other ventilation indicators was observed in our analyses.

Exploring more microscopic pathophysiological mechanisms, we can use the theory of delaying the overactivation of the respiratory muscle metaboreflex to explain the pathway by which IMT optimizes overall exercise capacity. During increased exercise load, COPD patients with severe airflow obstruction are more prone to develop or worsen dynamic lung hyperinflation, thereby limiting tidal volume expansion and worsening dyspnea (34, 35). At this point, the diaphragm, due to increased mechanical and metabolic demands, rapidly induces widespread sympathetic nerve discharge, leading to strong vasoconstriction in the lower limb vessels responsible for walking and “blood flow stealing”, causing premature ischemic fatigue of the extremities (36, 37). After a training cycle, IMT not only increases local motor unit recruitment but also upregulates the expression of oxidative enzymes in targeted muscle cells, greatly delaying the onset of exercise limitation. Recent mechanistic studies combining surface electromyography and dynamic MRI have confirmed that effective IMT can significantly reduce compensatory activation of accessory respiratory muscles (e.g., sternocleidomastoid) and improve diaphragmatic contractile efficiency and chest wall coordination (38).

Although this systematic review and meta-analysis provides clinically relevant evidence on IMT in COPD, several limitations should be acknowledged. First, the protocol was retrospectively registered on May 6, 2026, after data extraction had commenced. Therefore, this review cannot be considered prospectively registered, and exploratory subgroup analyses and meta-regression should be interpreted cautiously because of the potential risk of selective reporting. Second, although most included studies enrolled stable COPD patients, one study included participants in the post-AECOPD rehabilitation phase. Combining stable-phase and post-exacerbation recovery-phase participants may have increased clinical heterogeneity, because symptom burden, baseline functional status, and responsiveness to rehabilitation may differ between these populations. Sensitivity analyses excluding the post-AECOPD study did not materially change the direction of the main findings, but the small number of post-AECOPD studies precluded a reliable disease-phase subgroup analysis. Third, several included trials used multi-arm designs. To avoid double counting, we included only eligible IMT-specific arms and relevant comparator arms, split shared comparator groups when necessary, or combined conceptually similar IMT arms before pooling. Nevertheless, these decisions may still have introduced analytical complexity, particularly in trials comparing different IMT modalities or combining IMT with other rehabilitation components. Fourth, the included studies reported continuous outcomes using both change scores and post-intervention endpoint values. Although change scores were preferred and separate supplementary analyses were performed for change-score and endpoint-value studies, pooling these data forms may have contributed to statistical heterogeneity and should be considered when interpreting the pooled estimates. Fifth, dyspnea outcomes were measured using heterogeneous instruments, including Borg, mMRC, and TDI. Although all scales were harmonized so that positive SMD values indicated improvement and multiple dyspnea outcomes from the same study were selected or combined to avoid double counting, these scales capture different constructs, including daily-life dyspnea, exertional dyspnea, and transition-based clinical change. This heterogeneity may have diluted the pooled dyspnea effect and limits the clinical interpretability of the summary estimate. Sixth, the reliability of meta-regression and publication-bias assessment was limited by the small number of studies available for several outcomes and subgroups. Therefore, findings from comparator-based meta-regression, Egger’s tests, and funnel plots should be regarded as exploratory rather than confirmatory. The absence of clear funnel-plot asymmetry or statistically significant Egger’s tests should not be interpreted as definitive evidence against publication bias. Finally, a formal GRADE assessment was conducted to evaluate the certainty of evidence for the main outcomes. The certainty of evidence was rated as low for PImax and 6MWD, and very low for dyspnea and FEV₁, mainly because of serious inconsistency, imprecision, risk-of-bias concerns, heterogeneity in outcome measurement, and the influence of individual studies on some pooled estimates. Therefore, although IMT appears to improve inspiratory muscle strength and may improve exercise tolerance, the overall confidence in the magnitude of these effects remains limited.

## 5 Conclusion

Current systematic evidence suggests that structured IMT may be a feasible lower-load option for COPD patients, including those unable to participate in standard pulmonary rehabilitation. IMT holds potential to improve inspiratory muscle strength and exercise tolerance, but further high-quality studies are needed to confirm its optimal application across different patient subgroups. Future research urgently needs to deploy high-quality, head-to-head, multi-country comparative designs with long-term adherence assessment and comparison of different resistance directions (inspiratory versus expiratory bidirectional interaction). Furthermore, the incorporation of digital smart devices with automated resistance adjustment and real-time feedback monitoring is expected to drive large-scale respiratory muscle rehabilitation systems toward precision and digitalization for patients with severe airway obstruction.

## Data Availability

The original contributions presented in the study are included in the article/Supplementary material, further inquiries can be directed to the corresponding author.
