# Supplementary material for: Effects of inspiratory muscle training on inspiratory muscle strength and exercise tolerance in patients with COPD: a meta-analysis and systematic review
Source: Front Med (Lausanne). 2026 Jun 29;13:1855676. doi: 10.3389/fmed.2026.1855676 (PMC13358839; doi:10.3389/fmed.2026.1855676)
Supplement: Supplementary file 1 [file Supplementary_file_1.docx]

Supplementary Table S1. Study-Level Risk of Bias Assessment (RoB 2.0)

| **Study** | **Randomization Process** | **Deviations from Intended Interventions** | **Missing Outcome Data** | **Measurement of the Outcome** | **Selection of Reported Result** | **Overall Risk** |
| --- | --- | --- | --- | --- | --- | --- |
| Beaumont 2015 [13] | Low | Some concerns | Low | Low | Low | Some concerns |
| Chuang 2017 [14] | Low | Low | Low | Low | Low | Low |
| Langer 2018 [15] | Low | Some concerns | Low | Low | Low | Some concerns |
| Xu 2018 [16] | Some concerns | Low | Low | Low | Low | Some concerns |
| Schultz 2018 [17] | Low | Some concerns | Low | Low | Some concerns | Some concerns |
| Beaumont 2018 [18] | Low | Low | Low | Low | Low | Low |
| Cutrim 2019 [19] | Low | Low | Low | Low | Low | Low |
| Saka 2021 [20] | Low | Low | Low | Low | Low | Low |
| Tounsi 2021 [21] | Low | Low | Low | Low | Low | Low |
| Wu 2024 [22] | Low | Low | Low | Low | Some concerns | Some concerns |
| Dosbaba 2025 [23] | Low | Low | Low | Low | Low | Low |
| Elsayed 2026 [24] | Some concerns | Low | Low | Low | Some concerns | Some concerns |
| Huang 2025 [25] | Low | Low | Low | Low | Low | Low |


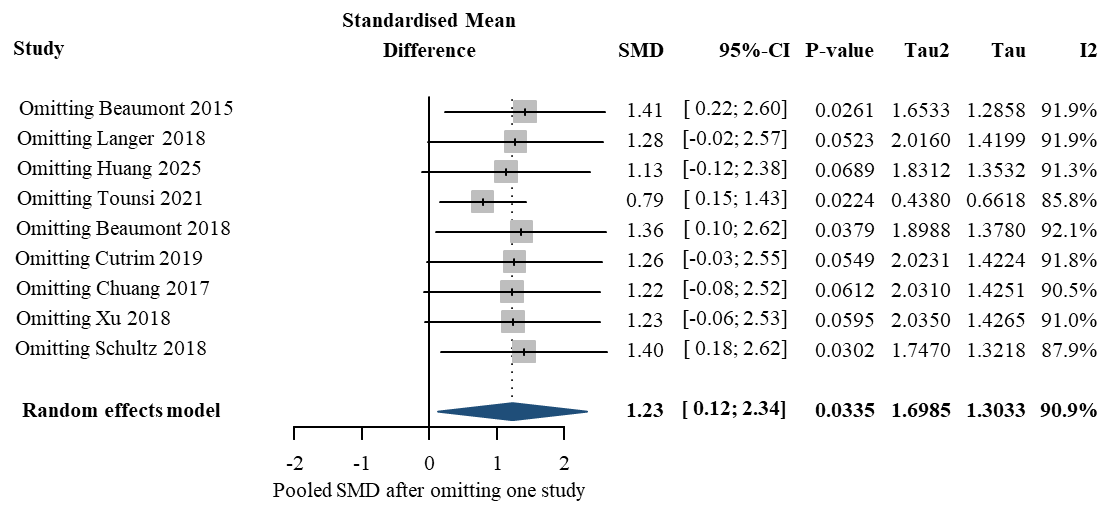


**Figure S1. Leave‑one‑out analysis of PImax**


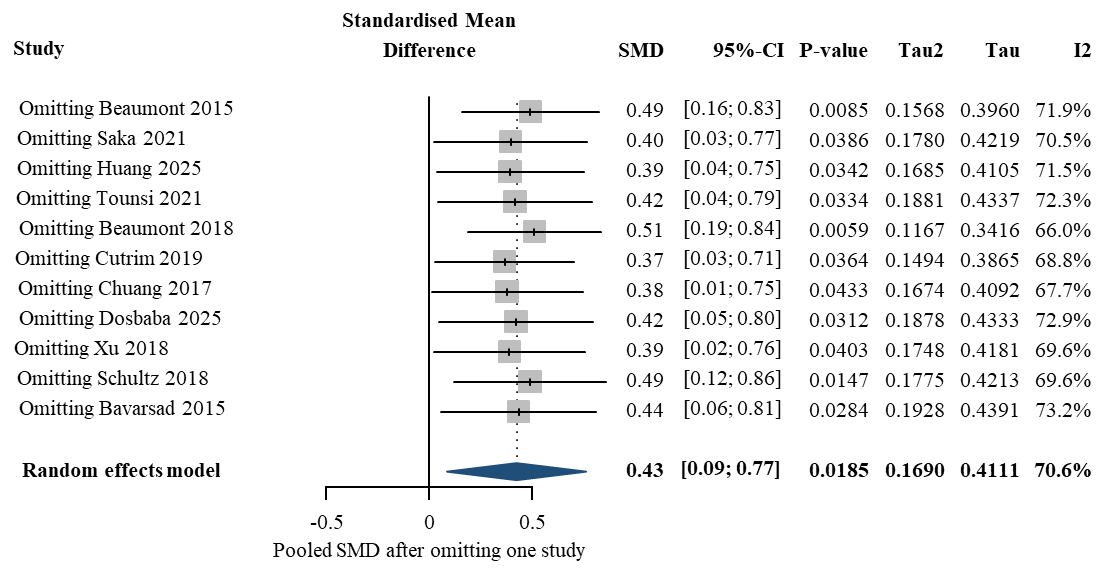


Figures S2**. Leave‑one‑out analysis of 6MWD**


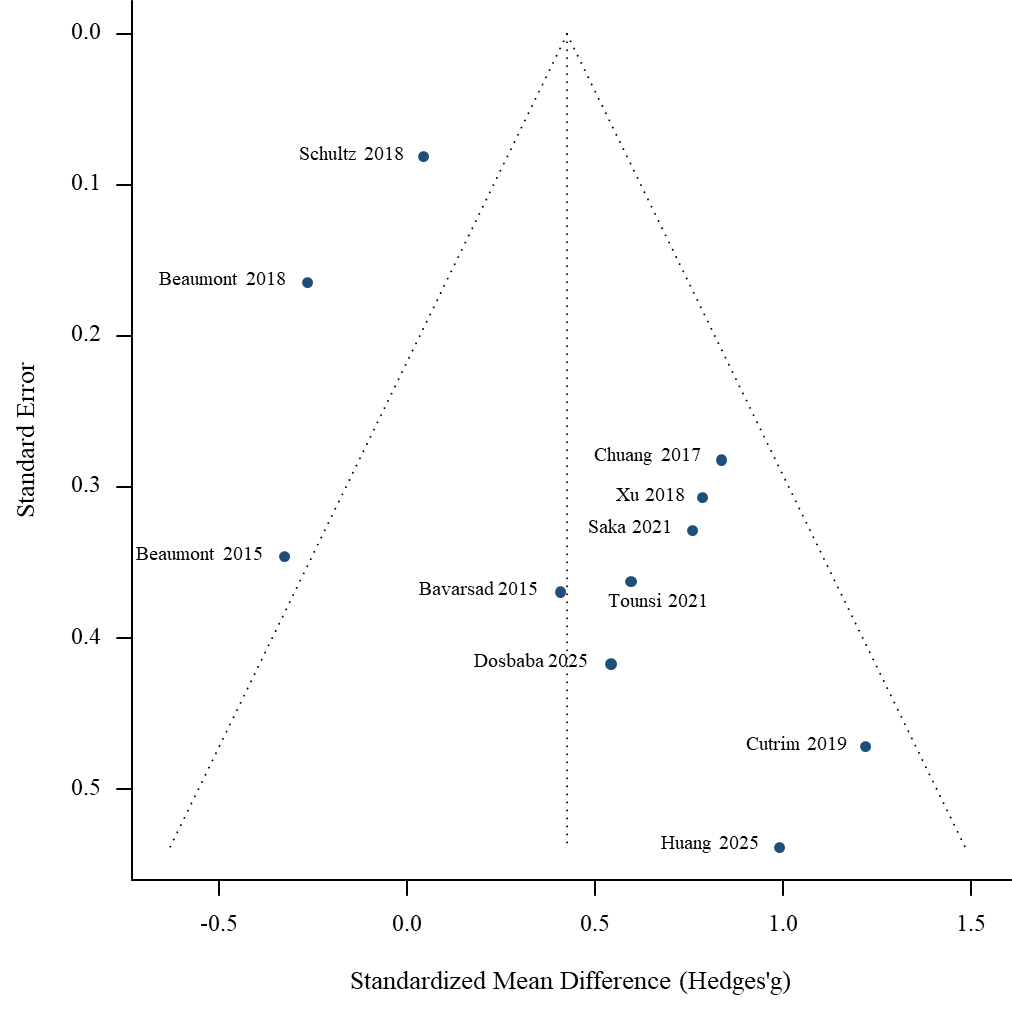


Figures S3**. Funnel plot of 6MWD**
