# Supplementary material for: Effects of inspiratory muscle training on inspiratory muscle strength and exercise tolerance in patients with COPD: a meta-analysis and systematic review
Source: Front Med (Lausanne). 2026 Jun 29;13:1855676. doi: 10.3389/fmed.2026.1855676 (PMC13358839; doi:10.3389/fmed.2026.1855676)
Supplement: Supplementary file 3 [file Table_2.docx]

Supplementary Table S2. GRADE summary of findings for the main outcomes

| **Outcome** | **No. of studies / participants** | **Effect estimate** | **Main reasons for downgrading** | **Certainty of evidence** |
| --- | --- | --- | --- | --- |
| PImax | 9 studies / 976 participants | SMD = 1.23, 95% CI 0.12 to 2.34 | Serious inconsistency (I² = 90.9%); imprecision due to wide confidence interval; some risk-of-bias concerns | Low |
| 6MWD | 11 studies / 1050 participants | SMD = 0.43, 95% CI 0.09 to 0.77 | Serious inconsistency (I² = 70.6%); comparator-dependent effects; some risk-of-bias concerns | Low |
| Dyspnea | 13 studies / 1170 participants | SMD = 0.33, 95% CI −0.11 to 0.77 | Serious inconsistency (I² = 77.6%); heterogeneity of Borg, mMRC, and TDI scales; imprecision because CI crossed the null effect | Very low |
| FEV₁ | 10 comparisons / 919 participants | MD = 0.14 L, 95% CI −0.17 to 0.44 | Serious inconsistency (I² = 79.4%); imprecision; substantial influence of Elsayed 2026 on effect size and heterogeneity | Very low |
