# Supplementary material for: Effects of inspiratory muscle training on inspiratory muscle strength and exercise tolerance in patients with COPD: a meta-analysis and systematic review
Source: Front Med (Lausanne). 2026 Jun 29;13:1855676. doi: 10.3389/fmed.2026.1855676 (PMC13358839; doi:10.3389/fmed.2026.1855676)
Supplement: Supplementary file 4 [file Table_3.docx]

Supplementary Table S3. Sensitivity analysis excluding the post-AECOPD rehabilitation-phase study

| **Outcome** | **Main analysis** | **Analysis excluding Huang 2025** | **Interpretation** |
| --- | --- | --- | --- |
| **PImax** | SMD = 1.23, 95% CI 0.12 to 2.34; P = 0.034; I² = 90.9% | SMD = 1.02, 95% CI 0.42 to 1.62; P = 0.001; I² = 91.3% | Effect remained favorable and statistically significant |
| **6MWD** | SMD = 0.43, 95% CI 0.09 to 0.77; P = 0.018; I² = 70.6% | SMD = 0.39, 95% CI 0.08 to 0.69; P = 0.012; I² = 71.6% | Effect remained favorable and statistically significant |
| **Dyspnea** | SMD = 0.33, 95% CI −0.11 to 0.77; P = 0.129; I² = 77.6% | Unchanged | Huang 2025 did not contribute data to this outcome |
| **FEV₁** | MD = 0.14 L, 95% CI −0.17 to 0.44; P = 0.190; I² = 79.4% | Unchanged | Huang 2025 did not contribute data to this outcome |
